# Distribution and densities of fish larvae species with contrasting life histories as a function of oceanographic variables in the deep-water region of the southern Gulf of Mexico

**DOI:** 10.1371/journal.pone.0280422

**Published:** 2023-02-13

**Authors:** Daudén-Bengoa Gonzalo, Sylvia Patricia Adelheid Jiménez-Rosenberg, Laura del Pilar Echeverri-García, María Ana Fernández-Álamo, Uriel Ordóñez-López, Sharon Z. Herzka

**Affiliations:** 1 Departamento de Oceanografía Biológica, Centro de Investigación Científica y de Educación Superior de Ensenada (CICESE), Ensenada, Baja California, México; 2 Instituto Politécnico Nacional, Centro Interdisciplinario de Ciencias Marinas (IPN-CICIMAR), La Paz, Baja California Sur, México; 3 Laboratorio de Invertebrados, Facultad de Ciencias, Universidad Nacional Autónoma de México, Ciudad de México, México; 4 Instituto Politécnico Nacional, Centro de Investigación y Estudios Avanzados, Unidad Mérida, Mérida, Yucatán, México; Universidade de Aveiro, PORTUGAL

## Abstract

We describe the larval occurrence and density of six fish species with contrasting life histories and examine their relationships with oceanographic variables during two seasons in the deep-water region (> 1000 m) of the southern Gulf of Mexico based on 12 cruises (2011–2018). Given that *Caranx crysos* adults are neritic, larval presence close to the continental shelf indicates offshore cross-shelf transport to oceanic waters, which likely leads to mortality. Generalized additive models indicated that *C*. *crysos* density was not related with oceanographic variables, while *Auxis* spp. (with neritic and oceanic adults) was related to wind speed, sea surface temperature, sea surface height, and surface chlorophyll a. The mesopelagic *Benthosema suborbitale*, *Notolychnus valdiviae* and *Bregmaceros atlanticus* were more abundant and broadly distributed, and higher density was found in conditions indicative of higher nutrient availability and productivity, suggesting greater feeding success and survival. The distribution of the epi- and mesopelagic *Cubiceps pauciradiatus* extended through the southern Gulf of Mexico, and was related to wind speed, sea surface temperature, stratification and chlorophyll a. Our results suggest that the density of the neritic species in oceanic waters could be mediated by regional cross-shelf transport, while for oceanic species is linked with productivity.

## Introduction

Comparing the distribution, density and habitat characteristics of species with contrasting life history strategies can provide insight into how similar processes structuring biogeographical provinces and driving connectivity might relate differently with species with contrasting life histories. This would allow for the evaluation of the specific relationship between environmental conditions and how connectivity can affect survival during the early life stages. Marine fish species can be classified according to the habitat characteristics and depths where adults reside and spawn (e.g., [1, 2]). Neritic species are found from the coastline to the edge of the continental shelf (at depths < 200 m) and their spawning period is generally between spring and summer [2, 3]. Spawning normally occurs in the upper water column or in shallow demersal habitats such as coral reefs [4, 5], where wind-driven long-distance transport tends to occur along-shelf [6, 7], although cross-shelf transport can also cause larvae to be transported off the continental shelf toward deeper oceanic waters [8–10]. Larval stage duration in most neritic species is 16 to 40 days [11–13]. Species that live in oceanic waters can be classified in those that live in epipelagic (0–200 m), meso- (200–1000 m) and bathypelagic (1000–4000 m) waters [14]. While epipelagic species usually spawn between spring and summer [3, 15], mesopelagic species mostly spawn throughout the year, although some present one or two spawning peaks (April-May, August-September; [2, 3]). However, fecundity is typically lower in oceanic species than in neritic species [16–18]. Meso- and bathypelagic species’ spawning occurs mainly above 500 m (see [19, 20]), and eggs are fertilized as they rise to a specific vertical position in the water column as a function of the lipid and protein content of the yolk sac [16, 21, 22]. The larval stage of many mesopelagic species lasts ~ 50 days [23, 24].

The survival and successful recruitment of the early life stages of fishes (eggs, larvae and juveniles) is crucial to sustain adult populations through time [25]. Ichthyoplankton distribution and density is dependent on adult population size, spawning regions and seasonality, and whether their environmental requirements are met [26, 27]. In addition, due to the larval weak swimming ability, mainly until notochord flexion is completed [28, 29], some can be passively transported horizontally over tens to hundreds of kilometres [10, 27, 30], during which time they are exposed to varying environmental conditions. Spatial and temporal variability in environmental conditions can lead to changes in the physiology and behaviour of ichthyoplankton, affecting larval survival and recruitment [31]. Understanding the spatial and temporal density of the larvae relative to oceanographic conditions allows for defining habitat requirements, as well as identifying habitat shifts caused by anthropogenic impacts such as oil spills, overfishing and climate change, all of which affect survival and recruitment during the early life of marine fishes [32–34].

The Gulf of Mexico (GoM) is a semi-enclosed large marine ecosystem in which the central oceanic region encompasses 70% of its total area [35]. The circulation is dominated by mesoscale features including the Loop Current (LC), detached LC anticyclonic eddies (LCEs), non-LC anticyclonic eddies (AC) and cyclonic eddies [7, 36, 37]. The LC connects the Caribbean Sea to the GoM through the Yucatan Channel, and becomes the Gulf Stream in the Atlantic Ocean once it passes through the Straits of Florida [38]. LCEs travel westward in the central GoM over time periods ranging from months to one year [37, 39]; when they reach the slope and the continental shelf their energy dissipates [40]. In the LC and warm core eddies (LCEs and AC eddies), the pycnocline deepens due to the anticyclonic circulation and the productivity is lower [41]. The southern GoM (sGoM) is characterized by a semipermanent cyclonic eddy in the Bay of Campeche (BoC; South of 22°N; [36]), which provides nutrients to the surface due to a shallowing of the pycnocline [42]. Coupled with regional upwelling, the BoC exhibits higher productivity than the central gulf [8, 43]. While the circulation and productivity of the oceanic GoM is mainly influenced by mesoscale features, the continental shelf is primarily affected by wind direction and speed, seasonal surface and water column temperature variations [44, 45], salinity shifts and nutrient inputs driven by river discharges (e.g., Mississippi river in the north and the Grijalva-Usumacinta riverine system in the south). In addition, the GoM’s circulation and environmental conditions in the surface layer are heavily influenced by atmospheric cold fronts during fall and winter [46, 47], and by tropical storms and hurricanes during summer and fall [48]. The circulation and environmental conditions of the oceanic and neritic regions may cause changes in the adult spawning areas and the spatial distribution of the suitable habitat of the larvae, affecting their distribution and density [33, 49].

Larval fish studies in the GoM that include species with contrasting life histories have mainly focused on larval fish assemblages, in which groups of species are considered an entity [50, 51]. For example, Muhling *et al*. [52] examined data from 20 years of SEAMAP surveys (from the coast to 2000 m) in the northern GoM and found an increase in the density of oceanic families and a decrease in neritic families through time. Inner shelf species’ larval density was positively related with shrimp-trawling effort and with the Mississippi River outflow, while those of outer shelf species was positively correlated with mean SST and with plankton density, which influences larvae survival through food availability. Studies of fish larvae in the deep-water region have also focused on commercially important species such as tunas and billfishes (Scombridae and Istiophoridae; [49, 53, 54]) as well as snappers and runners (Lutjanidae and Carangidae; [18, 55, 56]), but these studies have focused on the US Exclusive Economic Zone (EEZ) and have not been extended to the southern gulf. However, a comparative approach that examines the distribution and habitat characteristics of species with different life histories in the GoM’s deep-water region is lacking.

For the Mexican EEZ, Sanvicente-Añorve *et al*. [57] identified four assemblages in BoC’s shelf and oceanic regions, which varied spatially and seasonally. They suggested that adult habitat, seasonality and transport processes led to distinct ichthyoplankton assemblages. Compaire *et al*. [10] used backward-tracking particle experiments to explain the presence of coastal and neritic species in the oceanic waters of the western GoM, thus providing insight into the transport of larvae from the continental shelf to the deep-water region. Daudén-Bengoa *et al*. [51] suggested that adult distribution and spawning drove the species composition and distribution of larval assemblages of lanternfishes (Myctophidae) in the deep-water region, rather than larval transport. Therefore, this study will allow to comprehend how same environmental conditions correlate differently depending on the species’ contrasting life histories.

The aim of this study was to describe the larval distribution and density relative to environmental conditions of six species with contrasting life history strategies and adult habitats, based on surveys performed in the deep-water region of the sGoM. The relationship with *in situ* and satellite-based oceanographic parameters during two seasons (spring and early summer, and late summer and autumn) was examined based on the analysis of an extensive dataset integrated from 12 cruises performed between 2011 and 2018. We hypothesized that oceanic mesopelagic species would be broadly distributed, and that their density would be related with variables indicative of mesoscale cyclonic and anticyclonic eddies. In contrast, the distribution of neritic species should be more limited to stations close to the edge of the continental shelf in areas where cross-shelf transport is observed, and there should not be a relationship with oceanographic variables. Since mesopelagic species usually spawn throughout the year, temporal variability in density between seasons should not be observed, unlike for neritic species in which spawning seasonality predominates.

## Material and methods

### Sampling design

**Fig 1:**
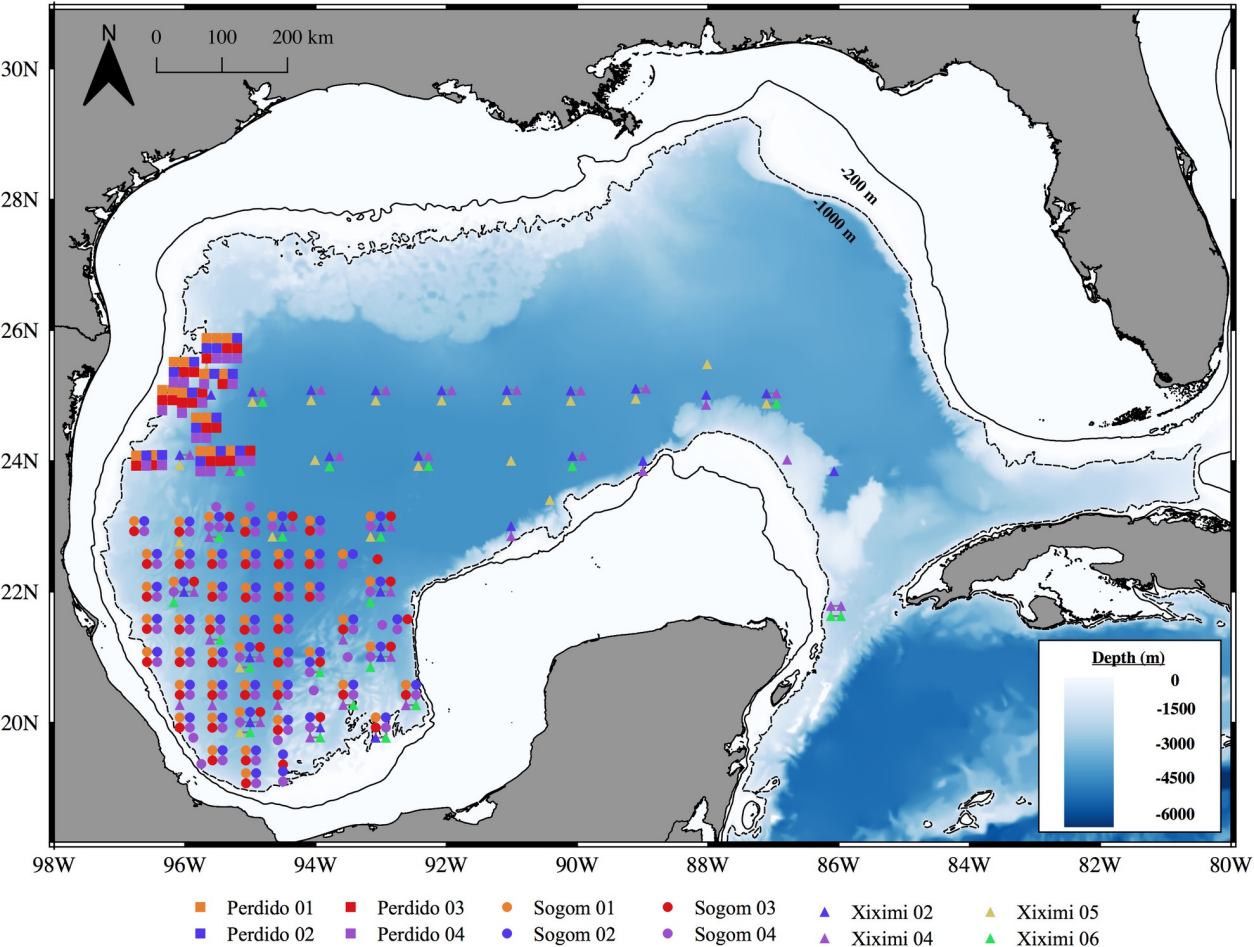
Ichthyoplankton sampling stations covered during cruises held between 2011–2018 in the southern Gulf of Mexico. PERDIDO (squares), SOGOM (circles) and XIXIMI (triangles) cruises. To allow for the visualization of multiple samples collected at a given station, their location is represented as a grid. Yucatan Channel stations were only sampled in season II. Continuous and dashed lines represent the 200 m and the 1000 m isobaths, respectively.

Samples were collected during 12 oceanographic cruises (Table 1), XIXIMI (18 to 25°N, 86 to 97°W), SOGOM (18 to 23°N, 86 to 92°W) and PERDIDO (24 to 26°N, 95 to 97°W) on board the R/V *Justo Sierra* and covered different regions of the sGoM’s deep-water region with some spatial overlap (Fig 1). The cruises were all conducted under a single multi-institutional research project funded by the Mexican National Council for Science and Technology—Mexican Ministry of Energy—Hydrocarbon Fund (project 201441). Cruises were classified to season I comprising spring to early summer (April to July) and season II encompassing late summer to autumn (August to October). This division was based on the increasing stratification that is observed in late summer and early fall compared to spring and early summer, and allowed for a balanced pooling of the sampling effort (n = 6 cruises per season). It is also supported by significant differences (Wilcox Test, p<0.05) between seasons in all oceanographic variables selected for the statistical analysis (Table 4). Although our approach is not conducive to examining larval distribution over the temporal scale of a specific cruise, the seasonal pooling of density data for cruises conducted over several years (see [23, 58–60]) allowed us to include more data and obtain more robust statistical analyses that reflects a larger sampling effort that encompasses interannual variability.

**Table 1:** Cruises in which larval fish sampling surveys were conducted presented in chronological order.

Cruise	Dates	Season	No. of stations
**XIXIMI 02**	01–15 July 2011	I	34
**SOGOM 01**	3–27 June 2015	I	51
**XIXIMI 04**	27 August—16 September 2015	II	34
**PERDIDO 01**	12–21 May 2016	I	13
**XIXIMI 05**	10–24 June 2016	I	32
**SOGOM 02**	31 August—20 September 2016	II	48
**PERDIDO 02**	28 September—7 October 2016	II	12
**SOGOM 03**	21 April—23 May 2017	I	51
**PERDIDO 03**	6–21 June 2017	I	13
**XIXIMI 06**	1–31 August 2017	II	36
**PERDIDO 04**	19 September—1 October 2017	II	12
**SOGOM 04**	29 August—22 September 2018	II	49

Cruises were classified to Season I (April-July) or II (August-October).

All stations were located beyond the edge of the continental shelf in the deep-water region (>1000 m) of the Mexican EEZ, and covered the sGoM and Yucatan Channel (Fig 1). Stations in the Yucatan Channel were only sampled in season II. Some of the stations of the SOGOM and XIXIMI cruises located south of 22°N share the same coordinates, but were covered during different seasons if held in the same year.

Ichthyoplankton samples were collected from 200 meters to the surface with oblique bongo tows fitted with 335 μm mesh nets. To estimate filtration volumes, net mouths were equipped with General Oceanics flowmeters. Each sample was fixed immediately either in 96% ethanol or 7% formalin buffered with sodium borate. Ichthyoplankton was sorted in the laboratory and identified to the lowest possible taxonomic level based on morphometric and meristic characteristics [2, 14]. Densities were standardized as larvae per 1000 m^-3^ of filtered water. To compare temporal differences between species and seasons, the average standardized density was calculated (mean ± standard deviation) by grouping the data for all cruises within each season.

### Species selection

A preliminary list of dominant species was generated, and then specific taxa with contrasting life histories and ecological or economic importance were selected. Six target species were then selected to include taxa with contrasting adult habitats and early life history characteristics, as well as families with ecological and economic importance (Table 2). The species were *Benthosema suborbitale* and *Notolychnus valdiviae* (lanternfishes; Myctophidae), *Bregmaceros atlanticus* (codlets; Bregmacerotidae), *Caranx crysos* (jacks; Carangidae), *Cubiceps pauciradiatus* (drift fishes; Nomeidae), and *Auxis* spp. (Scombridae; which includes larvae of *A*. *rochei rochei* and *A*. *thazard thazard*, known as bullet and frigate tuna, respectively). Both *Auxis* species were grouped to genus since their larvae cannot be distinguished morphologically and molecular identification is necessary [60, 61]. Nevertheless, both species share very similar distribution, habitat, depth range and spawning periods in the GoM [2, 3, 62].

**Table 2:** Species selected for GAM analysis, according to ecological and life history characteristics and/or commercial importance.

Species	Adult habitat	Adult depth range (m)	Spawning season	Ecological or commercial importance and estimates of global catch	Literature
***Caranx crysos***	(H) Neritic	0–100	Apr to May	Commercial fisheries	[2, 3, 18, 63–65]
	(V) Epipelagic		Aug to Sep	Sport / recreational fishing 5966 t (2011)	
***Auxis spp*.**	(H) Neritic and oceanic	10–100	Year around spawning	Commercial fisheries	[1–3, 15, 60–64, 66, 67]
			Peak: Jan to Apr & Jun to Aug	Sport / recreational fishing 172693 t (1996)	
	(V) Epipelagic				
***Bregmaceros atlanticus***	(H) Neritic and oceanic	50–2000	Year around spawning	Biogeographic indicator	[2, 3, 64, 68–70]
				Trophic link	
				6500 t (1973)	
	(V) Mesopelagic				
***Cubiceps pauciradiatus***	(H) Oceanic	50–820	Intermittent spawner	Prey for commercial species	[2, 3, 64, 71–75]
	(V) Epi—Mesopelagic				
				Potential future fishery	
			Peak: Dec to Apr	Trophic link (fish, birds, mammals)	
***Benthosema suborbitale***	(H) Oceanic	50–750	Year around spawning	Industrial food production	[3, 16, 23, 64, 76, 77]
				Trophic link (fish, birds, mammals)	
	(V) Epi—Mesopelagic				
			Peak: May to Jul	Biogeographic indicator	
***Notolychnus valdiviae***	(H) Oceanic	25–800	Year around spawning	Industrial food production	
	(V) Epi—Mesopelagic			Trophic link (fish, birds, mammals)	
			Peak: Jan to Mar & Jul to Nov		
				Biogeographic indicator	

For descriptive purposes, adult habitats are divided into their horizontal (H: neritic, oceanic) as well as their vertical habitat (V: epipelagic, mesopelagic, bathypelagic).

### Oceanographic variable selection and processing

Hydrographic parameters were characterized through *in situ* measurements and remote sensing. *In situ* variables including temperature, salinity and fluorescence were measured at each station with a SBE 9Plus CTD equipped with a Seapoint chlorophyll a (chl a) fluorometer. Mean water column temperature over the sampling depth range (0–200 m) as well as temperature at 200 m were also calculated because it is a key parameter linked to the development and growth of fish larvae [78, 79]. The 0–200 m mean salinity can be used to detect freshwater transport to offshore waters [65, 80]. The depth of the 17°C isotherm and nitracline depth (density 25.3 mg m^-3^) are considered proxies of mesoscale structures (LCEs, non-LC AE and CE) in the GoM [81, 82]. Stratification (J, zero for a well-mixed layer and increases with stratification; [83]) was used as an *in situ* indicator of vertical mixing, since it has been related to nutrient and prey availability in the euphotic zone in oceanic waters [84, 85].

Sea surface temperature (SST; product id 010_005, 0.25° x 0.25° spatial resolution, gap-free gridded data after validation process) and sea surface height (SSH; product id 008_047, 0.25° x 0.25° spatial resolution, gap-free gridded data after validation process) were also used as indicators of mesoscale features. Sea surface chl a concentration (product id 009_082, ~ 0.04° x 0.04° spatial resolution, gap-free gridded data after validation process) was used as proxy of phytoplankton biomass and indicator of food availability [82, 86]. Satellite variables were obtained from the E.U. Copernicus Marine Service Information (CMEMS; [87]) (http://marine.copernicus.eu/). Spatial data for SST, SSH and sea surface chl a corresponded to the date in which each station was sampled. Wind surface speed (WS; ERA5 dataset, 31 km spatial resolution, gap-free gridded data after validation process) was downloaded from COPERNICUS (https://cds.climate.copernicus.eu/; [88]) and was calculated from wind surface speed vectors (u, v) using the coordinates for each station. WS was used as an indicator of turbulence, which has been related to larval feeding success [89, 90]. Data were averaged for the 4 days prior to the sampling date, because wind events such as northern fronts and southern winds in the GoM, are usually observed over the time periods of 3 to 7 days. Additionally, the bathymetry from ETOPO1 1 Arc-Minute Global Relief Model [91] was used since species from coastal and oceanic habitats were compared. Additionally, the location of each station (latitude and longitude) was added as a smoothed interaction term to consider spatial effects and account for spatial autocorrelation [54, 92]. Outliers were visualized using Cleveland dotplots [93] and only the variables that met the requirements of low correlation (Spearman ρ, r < 0.70) and a variance inflation factor (VIF) lower than 3 [93] were included in the statistical analyses: Mean salinity 0–200 m, SSH, SST, stratification, surface chl a and WS (Table 4).

### Data and statistical analysis

Temporal differences in the densities of larvae and oceanographic variables were compared with a Kruskal-Wallis (rank sums; α = 0.05) as an alternative to one-way analysis of variance (ANOVA). This non-parametric method does not assume a normal distribution, which was required since densities and oceanographic variables did not follow a normal distribution (Shapiro-Wilk normality test performed), and some of the variables did not show homogeneity of variance (Bartlett test performed).

Generalized additive models (GAMs) were used to examine the relationship between oceanographic conditions and the standardized density of each species. GAMs, an extension of generalized linear models, are a nonparametric and nonlinear regression technique that do not require *a priori* specifications of the functional relationship between the response and predictor variables [94, 95]. In the GAM equation:

$$E[y]=g^{-1}\left(\beta_{0}+\sum_{k}S_{k}(x_{k})\right)$$

*E[y]* equals the expected values of the response variable (standardized density of each species), *g* represents the link function, *β*_*0*_ equals the intercept, *x* represents each of *k* explanatory variables, and *S*_*k*_ represents the smoothing function for each of the explanatory variables.

Since all oceanographic variables were significantly different between seasons (Wilcox Test, p<0.05; Table 4) the GAMs were performed separately for each season. This strategy was chosen as it also allows for the characterization of the potential habitat distribution for the target species on a seasonal basis (see [34]). For comparative purposes, GAMs using season as a categorical variable and as a categorical smoothing term were also performed (S1 Table, S1 Fig). However, the percent of variance explained were generally lower than the GAMs with the split seasonal data base. Models were built with a logarithmic link function and using smoothing splines. The smoothing splines flexibility is associated with the degrees of freedom (1 = linear model; > 1 = nonlinear model; Wood, 2006 [96]). The Tweedie distribution (part of the exponential dispersion model family) was used since the response variable (standardized densities) had a high proportion of zeros and were non-negative [97, 98]. The percentage of zero densities for each response variable is reported in the result section (Table 5). The power value of the model, which indicates whether the data exhibited a gamma-like (power value close to 1) or a Poisson-like (power value close to 2) distribution is also reported. The Restricted (Residual) Maximum Likelihood (REML) was used as a smoothing parameter estimation method. To avoid overfitting in the model, variables were limited to a maximum 4 *k* parameters excepting latitude and longitude. The gam function from the “mgcv” package was used for the models and the gam.check function for model validation [99].

A stepwise manual backward procedure to identify the variables that had no effect on the explanatory variable (p > 0.05) was used (e.g., [54, 100]). This process was halted when all explanatory variables were significant (p < 0.05). Deviance explained was used as a measurement of goodness of fit [95]. If no variables were significantly related with a species’ density, or the explained deviance in the final model was lower than 10% (Deviance explained < 0.1) the model was discarded. Once the models were defined, the relative importance of each oceanographic variable was determined by examining the differences in the model with and without the variables by removing them one by one [54]. To assess the latter, the change in explained deviance and in AIC (Akaike information criteria) were used. In order to determine if the relationship between the density and the environmental variable was linear, the estimates of degrees of freedom were examined. The R–project 3.4.1 [101] statistical program was used for all analyses.

## Results

### Larval standardized distribution and density

The lanternfish *N*. *valdiviae* showed the highest larval density in both seasons, while the neritic species *C*. *crysos* presented the lowest (Table 3). When comparing between seasons, the mean density of *C*. *crysos* was almost three times higher in season II, although statistical differences were not found. *C*. *pauciradiatus* presented significantly higher mean larval density in season I, and *B*. *atlanticus* in season II. The density of the lanternfishes (*B*. *suborbitale* and *N*. *valdiviae*) did not differ between seasons.

**Table 3:** Mean seasonal density (larvae 1000 m^-3^) for cruises (6 per season) and total density conducted between 2011 and 2018 in the deep-water region of the southern GoM.

	Mean density ± Std. Deviation	
	Season I (April-July)	Season II (August-October)
*Caranx crysos* (p = 0.340)	0.25 ± 1.34	0.62 ± 2.52
	0.45 ± 2.06	
*Auxis* spp. (p = 0.898)	4.07 ± 10.20	4.09 ± 11.06
	4.08 ± 10.66	
***Bregmaceros atlanticus* (p** = **0.019)**	4.41 ± 8.70	5.77± 12.59
	5.14 ± 10.98	
***Cubiceps pauciradiatus* (p = 0.045)**	9.59 ± 21.90	5.75 ± 12.56
	7.67 ± 17.95	
*Benthosema suborbitale* (p = 0.068)	12.10± 16.98	9.26± 14.96
	10.57 ± 15.96	
*Notolychnus valdiviae* (p = 0.175)	20.97 ± 26.47	15.51 ± 17.30
	18.02 ± 22.13	

Results of one-way ANOVAs are reported between parentheses. Species with significant differences in mean density between seasons are in bold.

**Fig 2:**
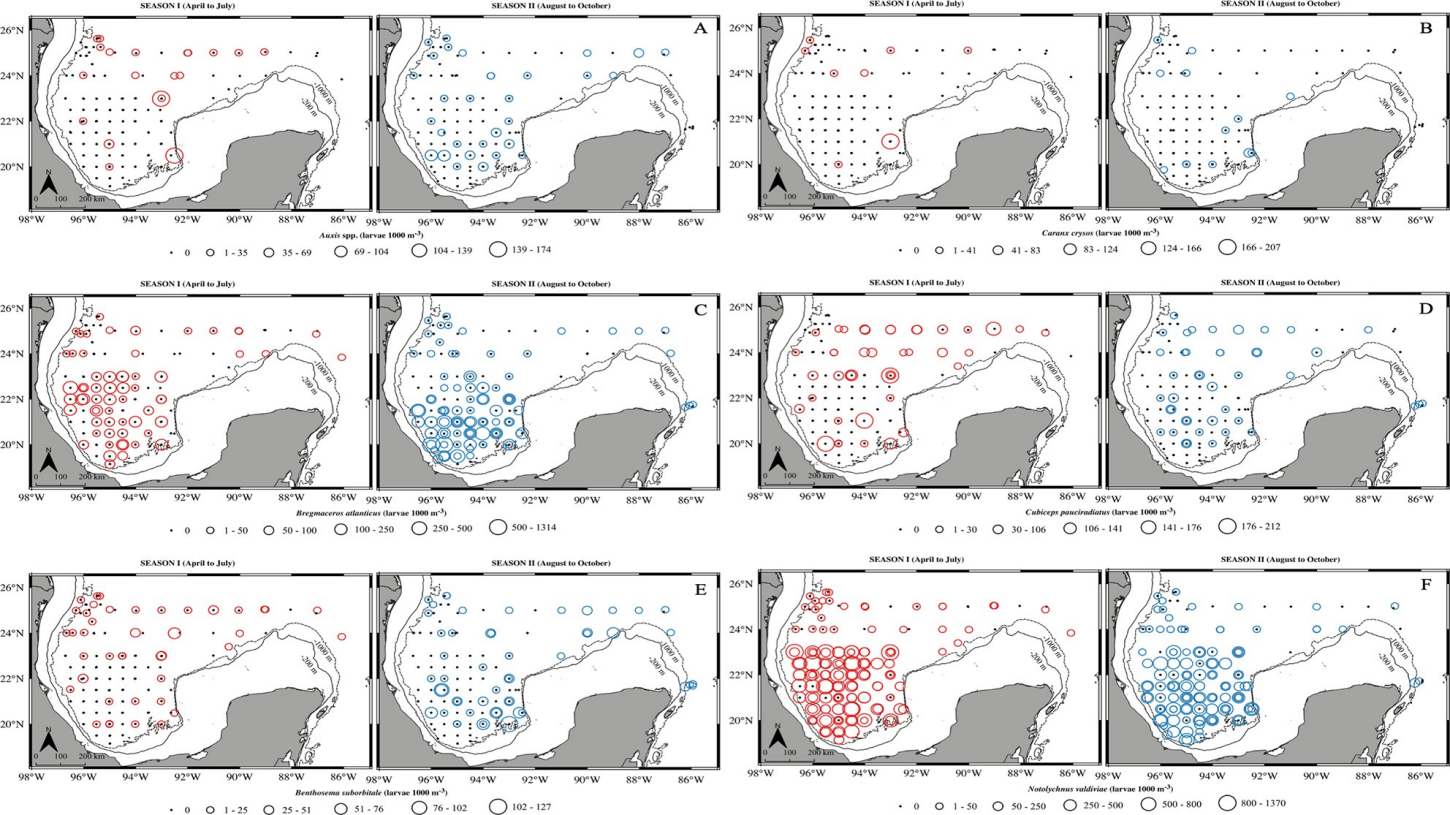
Season-specific spatial distribution of density of larval fish taxa. A: *Auxis* spp., b: *Caranx crysos*, c: *Bregmacero****s*** *atlanticus*, d: *Cubiceps pauciradiatus*, e: *Benthosema suborbitale*, f: *Notolychnus valdiviae*. First (second) column with red (blue) dots represents season I: April-July (season II: August-October). Circle size represents the density (larvae 1000 m^-3^) for a given location, with the scale adjusted for each species. Overlapping circles indicate samples from different cruises. Black dots indicate the absence of larvae. Continuous and dashed lines represent the 200 m and the 1000 m isobaths, respectively.

*Auxis* spp. was mostly distributed in the central gulf during season I in comparison to season II, when the highest densities were found in the BoC (Fig 2). *C*. *crysos’* distribution was mainly limited to stations closer to the continental shelf during season II, and some stations in the north-western and south-eastern gulf. However, in season I, larvae were found at some stations far from the continental shelf, in the central GoM. The distribution of *B*. *atlanticus* was patchy during both seasons, and high densities were found in the BoC although larvae were collected throughout the deep-water region. The spatial distribution of mesopelagic species was more homogeneous than that of neritic species, and species-specific seasonal variation was observed. While the spatial distribution of *B*. *suborbitale* and *N*. *valdiviae* was very similar between seasons, *B*. *suborbitale* was more abundant in the southern region of the study area in season II. The mesopelagic *C*. *pauciradiatus* showed the greatest density in almost all stations in the northern region and a higher degree of stations with larval absence were observed in the southern gulf during season I. In season II the distribution included more stations in the BoC.

### Generalized additive models

All oceanographic variables were significantly different between seasons (Wilcox Test, α = 0.05). Surface chl a and WS were significantly higher during season I (Table 4). Conversely, stratification, mean salinity, SST and SSH had significantly higher values in season II.

**Table 4:** Oceanographic variables comparison between seasons for cruises (6 per season) conducted between 2011 and 2018 in the deep-water region of the southern GoM.

	Mean ± Std. Deviation	
	Season I (April-July)	Season II (August-October)
**Wind speed (ms** ^**-1**^ **) (p < 0.001)**	4.70 ± 1.59	4.04 ± 1.46
**Surface chl a (mg m** ^**-3**^ **) (p = 0.014)**	0.11 ± 0.04	0.09 ± 0.04
**Mean salinity 0–200 m (psu) (p = 0.002)**	36.56 ± 0.08	36.60 ± 0.08
**SST (°C) (p < 0.001)**	28.40 ± 0.95	30.03 ± 0.41
**Stratification (J) (p < 0.001)**	1537.24 ± 395.69	1784.35 ± 282.62
**SSH (m) (p < 0.001)**	0.35 ± 0.09	0.41 ± 0.11

Kruskal-Wallis test results are reported between parentheses. Variables with significant differences (p <0.05) between seasons are in bold.

GAMs were constructed for five species: *Auxis* spp. (neritic and epipelagic), *B*. *atlanticus* (neritic and mesopelagic), and *B*. *suborbitale*, *C*. *pauciradiatus* and *N*. *valdiviae* (mesopelagic) (Table 5). The models for the neritic *C*. *crysos* had a very low explained deviance (< 0.1) in both seasons and were discarded. For *Auxis* spp., season I was the model with the highest explained deviance, and all models in season I presented a higher deviance explained than in season II (Table 5). In addition, station position also influenced in several models (Table 5). The relationship between the raw data of all species’ standardized densities and oceanographic variables are presented in S2 Fig.

**Table 5:** Final GAMs for each target species with significant environmental and spatial variables divided by season (Season I: April to July; Season II: August to October).

***Auxis* spp.**					
**SEASON I**	Zero (%): 63		**SEASON II**	Zero (%): 74	
***power = 1*.*30***	**DE = 47.3%**	**AIC = 421.03**	***power = 1*.*41***	**DE = 35.8%**	**AIC = 515.13**
Depth			Depth		
Stratification			Stratification	27.30	522.50
Salinity			Salinity		
SST	33.5	438.80	SST	30.90	520.04
SSH	43.7	424.86	SSH		
Chl	41.6	427.04	Chl	22.20	529.98
Wind speed	35.4	433.90	Wind speed	25.60	528.98
Lat, Lon			Lat, Lon		
***Benthosema suborbitale***					
**SEASON I**	Zero (%): 32		**SEASON II**	Zero (%): 41	
***power = 1*.*23***	**DE = 24%**	**AIC = 907.46**	***power = 1*.*38***	**DE = 23.5%**	**AIC = 978.28**
Depth			Depth		
Stratification	19.60	912.70	Stratification		
Salinity	12.50	923.18	Salinity		
SST			SST	16.50	990.97
SSH			SSH		
Chl	21.20	910.83	Chl	12.40	998.49
Wind speed	17.30	916.79	Wind speed	16.70	992.39
Lat, Lon			Lat, Lon		
***Bregmaceros atlanticus***					
**SEASON I**	Zero (%): 53		**SEASON II**	Zero (%): 45	
***power = 1*.*22***	**DE = 43.7%**	**AIC = 551.89**	***power = 1*.*35***	**DE = 22.7%**	**AIC = 867.52**
Depth			Depth		
Stratification	33.40	570.24	Stratification		
Salinity			Salinity		
SST	36.90	567.02	SST		
SSH			SSH		
Chl	41.80	559.86	Chl		
Wind speed			Wind speed		
Lat, Lon	18.90	566.68	Lat, Lon	22.70	867.52
***Cubiceps pauciradiatus***					
**SEASON I**	Zero (%): 50		**SEASON II**	Zero (%): 69	
***power = 1*.*38***	**DE = 45.6%**	**AIC = 686.81**	***power = 1*.*27***	**DE = 38.3%**	**AIC = 596.06**
Depth	35.70	700.77	Depth		
Stratification	40.40	692.78	Stratification		
Salinity			Salinity		
SST	34.90	704.81	SST	18.60	626.83
SSH	37.70	697.77	SSH		
Chl	40.20	693.01	Chl		
Wind speed			Wind speed	14.70	635.19
Lat, Lon			Lat, Lon		
***Notolychnus valdiviae***					
**SEASON I**	Zero (%): 18		**SEASON II**	Zero (%): 22	
***power = 1*.*37***	**DE = 44.4%**	**AIC = 1119.96**	***p = 1*.*37***	**DE = 18.8%**	**AIC = 1248.43**
Depth			Depth		
Stratification	41.50	1123.74	Stratification		
Salinity			Salinity		
SST	39.60	1126.85	SST		
SSH			SSH	17.50	1248.43
Chl			Chl		
Wind speed	37.20	1133.59	Wind speed		
Lat, Lon	24.80	1142.28	Lat, Lon	5.80	1268.66

DE and AIC columns indicates the model’s final values without the variable. Zero (%) indicates the percentage of zero values in the response variable (larval density).

**Fig 3:**
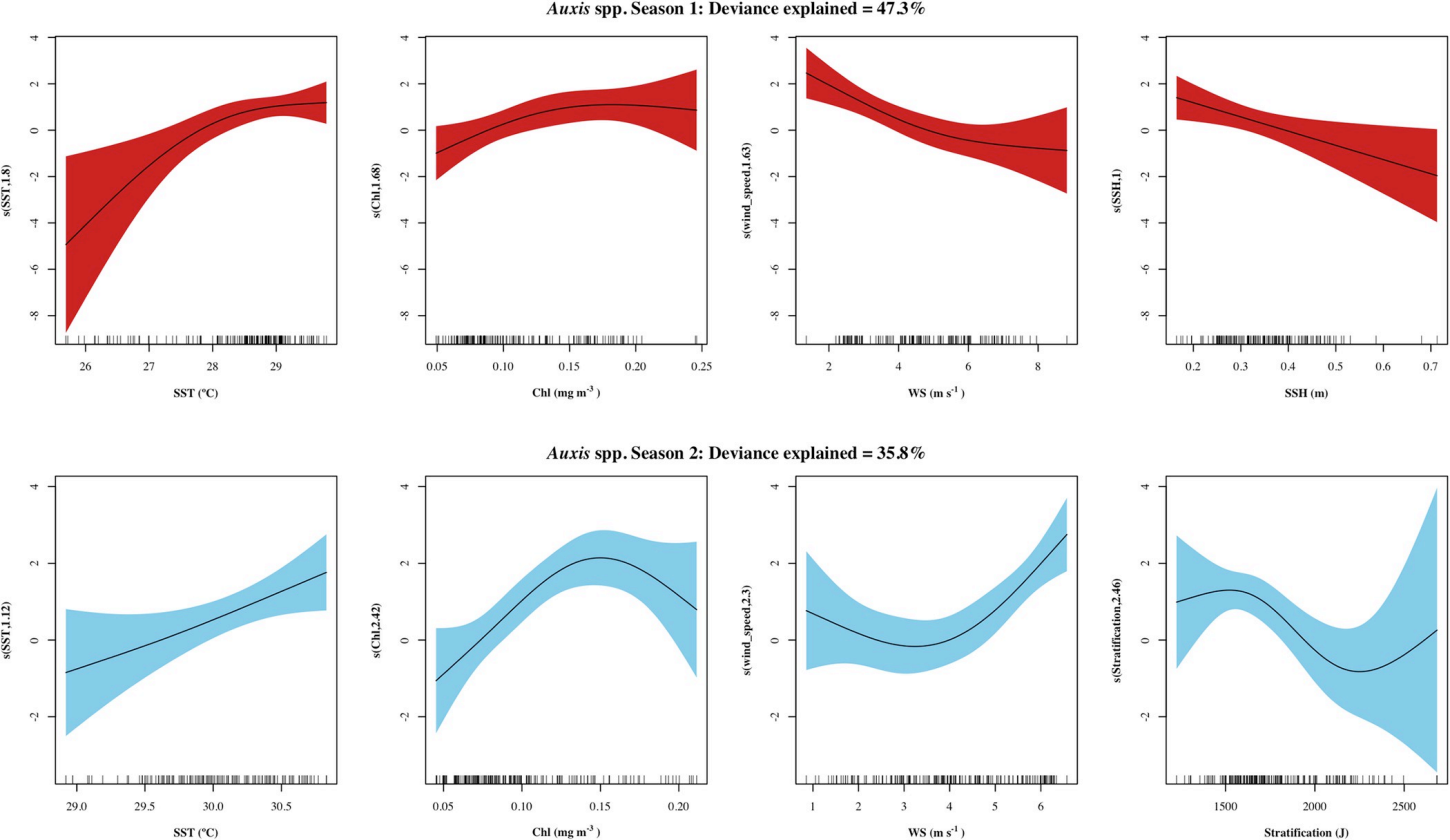
Response plots of the oceanographic variables’ additive effect on the density of *Auxis* spp. Season I (April-July) in red and season II (August-October) in blue. Smoothed values represented by a continuous line, and shaded color indicates 95% confidence intervals. variables with non-significant additive effects (p > 0.05) for a given season are not presented.

For season I, the final *Auxi*s spp. model included 4 oceanographic variables that were significantly related to larval density: SST, WS, chl a and SSH (listed in order of the highest explained deviance contributed to the model). For season II, 4 variables were included in the model (chl a, WS, stratification and SST); higher values of SST were related to higher densities, similar to what was observed for season II (Fig 3). A positive nonlinear relationship between density and chl a was observed for both seasons, although a decrease in density was observed in values > 0.15 mg m^-3^ in season II. Higher densities were related to lower WS in season I and to higher WS in season II. However, during both seasons most of the observations fell along intermediate WS values (3 to 6 m s^-1^). A linear negative relationship between SSH and density was observed for season I with most observations around intermediate to low values, while stratification was only significant in season II, with an increase in density at values < 1600 J, after which density decreased.

**Fig 4:**
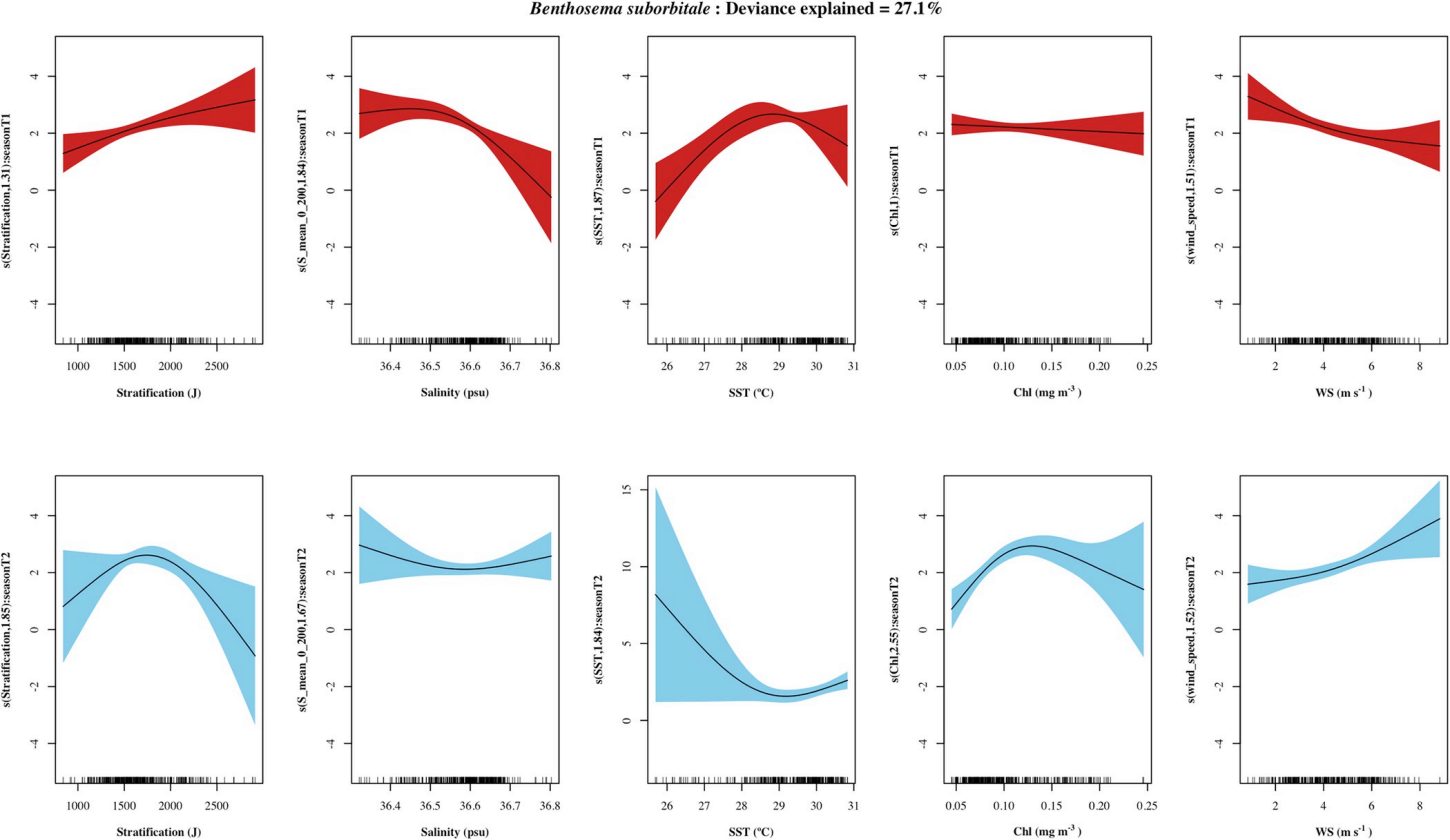
Response plots of the oceanographic variables’ additive effect on the density of *Benthosema suborbitale*. Season I (April-July) in red and season II (August-October) in blue. Smoothed values represented by a continuous line, and shaded color indicates 95% confidence intervals. Variables with non-significant additive effects (p > 0.05) for a given season are not presented.

*B*. *suborbitale*’s final model included 4 variables for season I (mean salinity, WS, stratification and chl a). For season II, the variables included in the models were only chl a, SST and WS. Most of the observations in relation with chl a were in low concentration values, however, while in season I a linear and negative relation was observed, in season II a positive non-linear relation was observed with a peak around 0.13 mg m^-3^ of chl a. WS showed an opposite relationship with density between seasons: it was negative and nonlinear in season I (Fig 4), with lowest densities between 5 to 8 m s^-1^, and with a linear and positive relationship for season II. Additionally, in season I mean salinity (the most powerful variable) was related with higher densities until a salinity of approximately 36.6 and stratification with values between 1500–2000 J. In season II SST was positively and non-linear related with B. suborbitale’s density.

**Fig 5:**
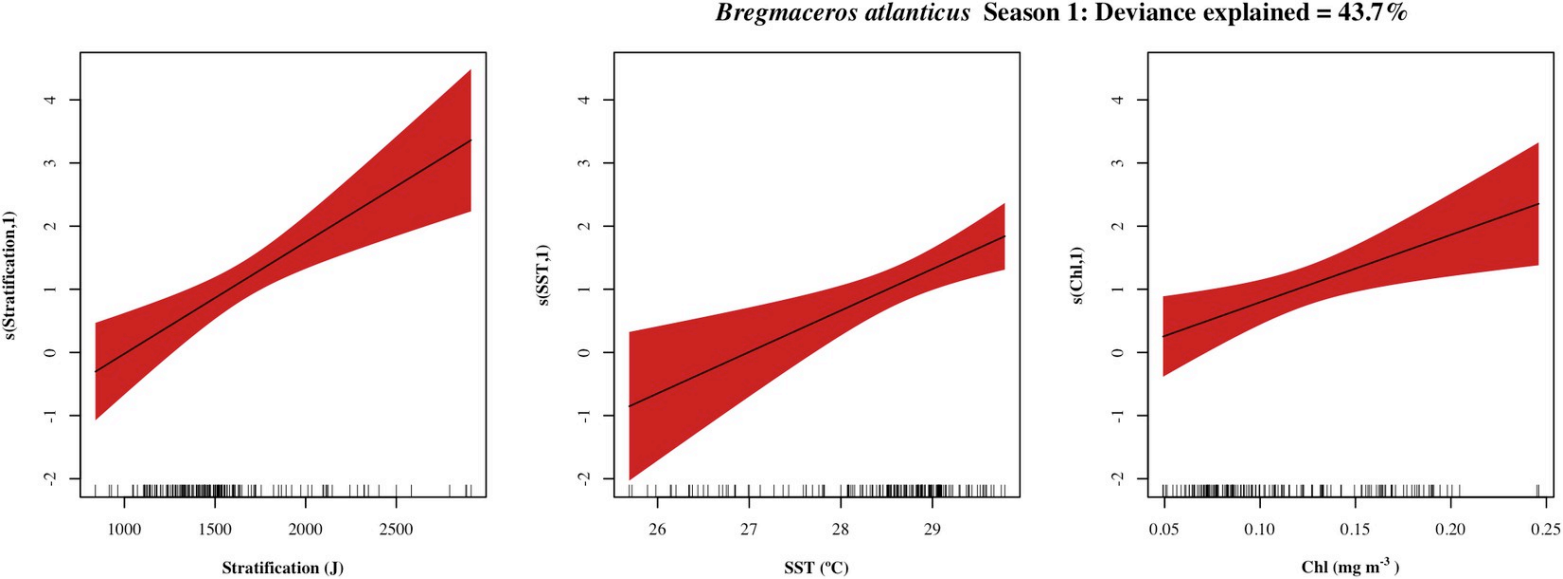
Response plots of the oceanographic variables’ additive effect on the density of *Bregmaceros atlanticus*. Season I (April-July) in red and season II (August-October) in blue. Smoothed values represented by a continuous line, and shaded color indicates 95% confidence intervals. variables with non-significant additive effects (p > 0.05) for a given season are not presented.

The density of *B*. *atlanticus* in season I was related to station location, stratification, SST and chl a, while in season II the explained deviance (DE = 22.7%) was only explained by station location. The three environmental variables were linear and positively related with larval density (Fig 5). Although, the greatest number of observations varied between the variables’ ranges. The majority of observations were in low stratification values (< 1600 J), while SSTs were above 28°C.

**Fig 6:**
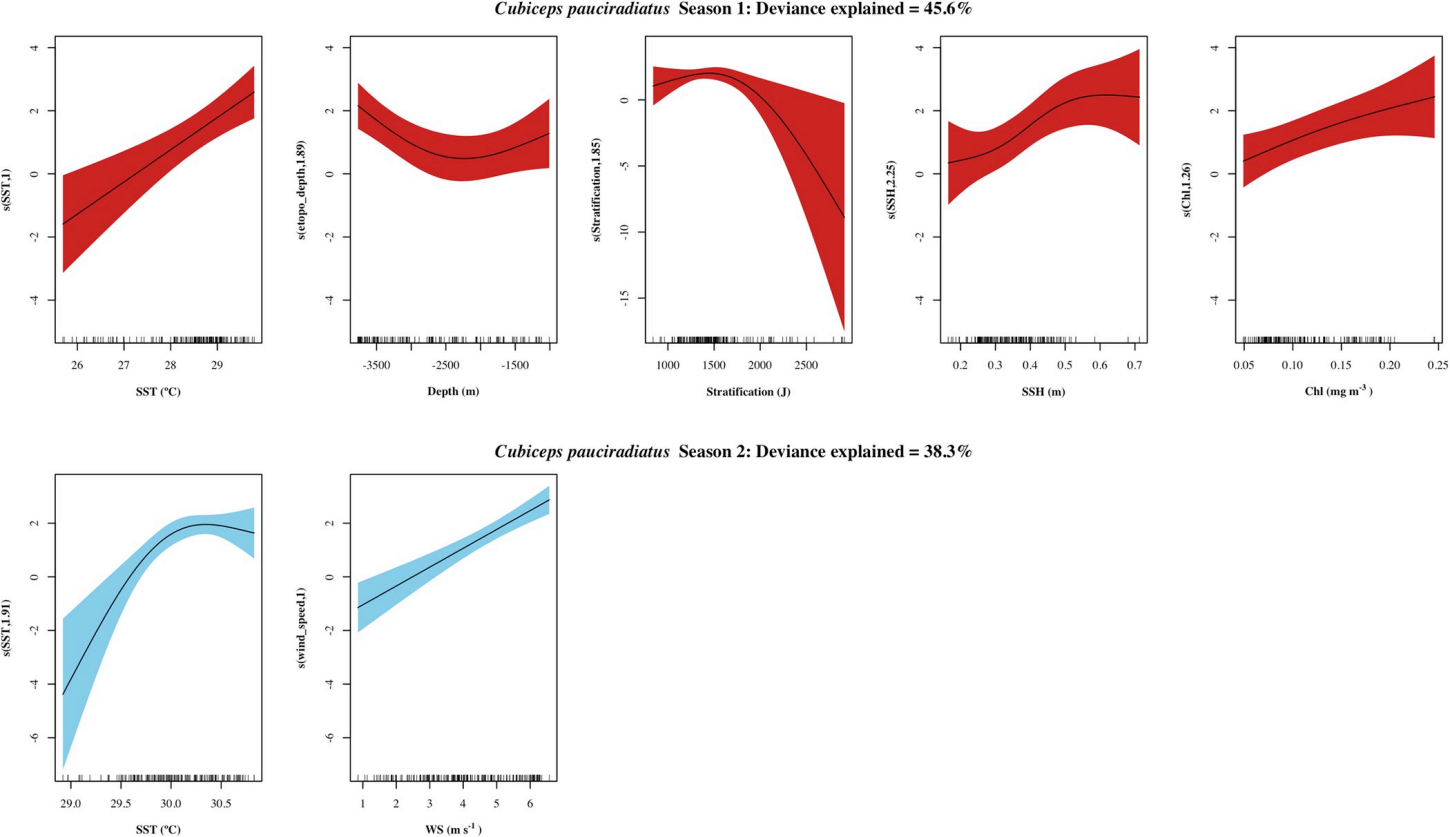
Response plots of the oceanographic variables’ additive effect on the density of *Cubiceps pauciradiatus*. Season I (April-July) in red and season II (August-October) in blue. Smoothed values represented by a continuous line, and shaded color indicates 95% confidence intervals. variables with non-significant additive effects (p > 0.05) for a given season are not presented. Y axis scale in season I plots were modified to allow for a better interpretation.

For *C*. *pauciradiatus*, SST was included in both models. For season I the explaining variables were SST, depth, SSH, chl a and stratification, while in season II WS and SST. A positive relationship between density and SST was found for both seasons (Fig 6); however, for season I the relationship was linear and the greatest densities were observed at temperatures higher that 28°C; and for season II maximum densities were > 30°C. Regarding the variables in season I, greater densities were found in deeper stations (< -3000 m), although densities increased in shallower waters than -2000 m deep. With regard to stratification, the highest densities were found at low values (1100 to 1600 J). SSH showed a nonlinear positive relationship with density in season I, with most of the observations found at low values (0.25 to 0.5 m), and a maximum at high SSH values (> 0.55 m), although this corresponded to very few observations. Thus, this relationship should be interpreted cautiously. Similar to previous models, larval density increased with chl a concentration. In season II WS was positive and linearly related with density.

**Fig 7:**
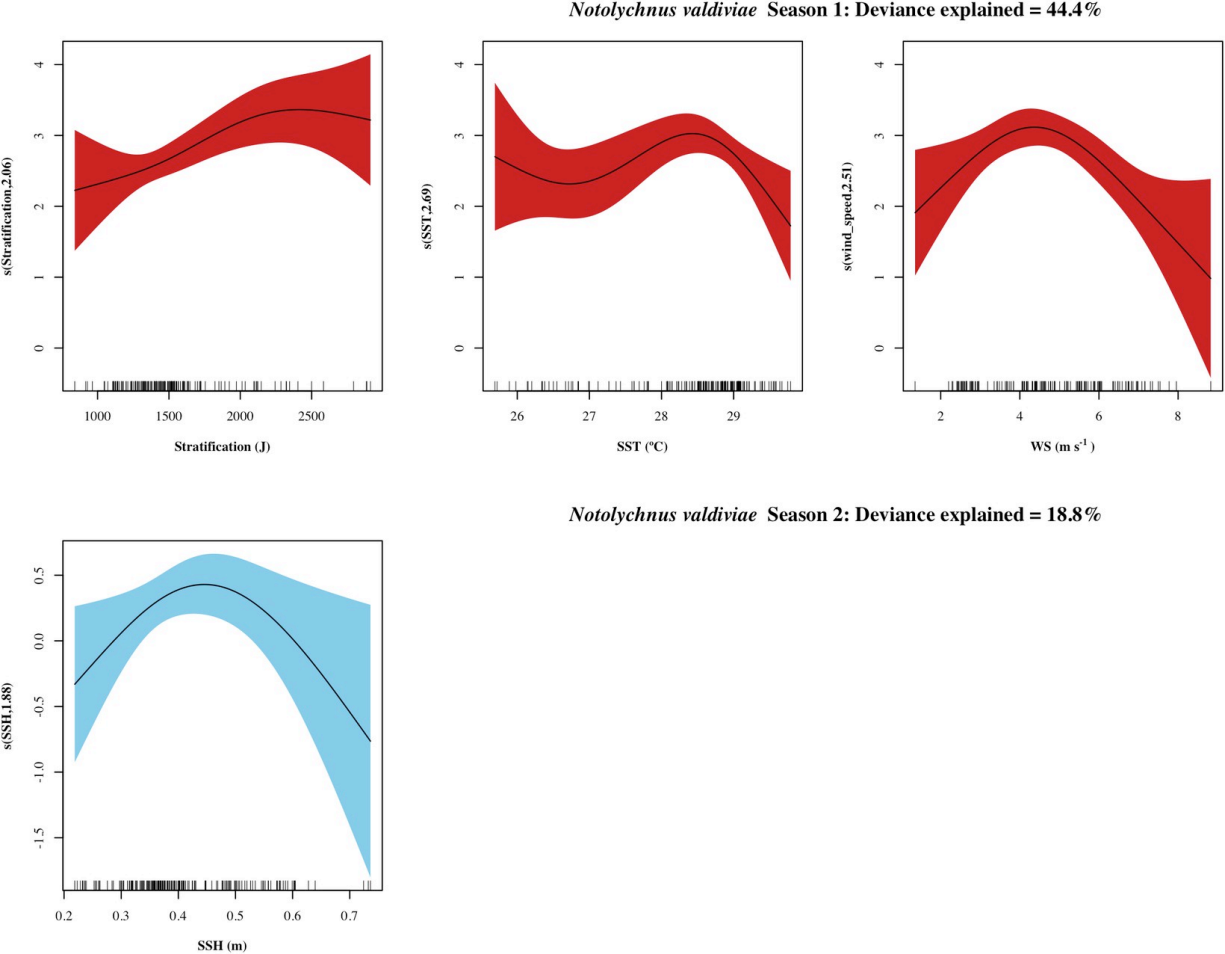
Response plots of the oceanographic variables’ additive effect on the density of *Notolychnus valdiviae*. Season I (April-July) in red and season II (August-October) in blue. Smoothed values represented by a continuous line, and shaded color indicates 95% confidence intervals. variables with non-significant additive effects (p > 0.05) for a given season are not presented.

For *N*. *valdiviae*, the variables retained for season I were station location, WS, SST and stratification, while for season II were station location and SSH. The relationship with stratification included was non-linear and positive, although most of the observations were found in values lower than 1700 J (Fig 7). An increase in density was found between 28 to 29°C of SST and a density peak was found at average WS values (4 to 5 m s^-1^). In season II a dome-like distribution was found in the relationship between density and SSH, with a maximum between 0.4 and 0.5 m.

## Discussion

In the deep-water region of the sGoM, the distribution and density of the larvae of fish species with contrasting life histories varied spatially and between seasons. Larvae of adults from neritic habitats were mostly captured closer to the slope and less abundant, compared to those from epi- or mesopelagic species that were more homogeneously distributed throughout the deep-water region. Additionally, the oceanographic variables that had a significant correlation with density, and thus some predictive power for delimiting their spatial and temporal distribution, varied among species. Although our approach is not conducive to examining larval distribution over the temporal scale of a specific cruise, the seasonal pooling of density data for cruises conducted over several years allowed us to include more data and obtain more robust statistical analyses.

### Spatial and temporal patterns

Spawning of the two neritic species occurs during March to April and June to August in *Auxis* spp. and April to May and August to September in *C*. *crysos* [2, 3, 62, 63], which is consistent with their presence during the two seasons considered in this study. These two species showed a more limited spatial distribution and lower densities than mesopelagic species, which agrees with our hypothesis. The adults of *C*. *crysos* live over the continental shelf at depths < 100 m [3, 64]. The distribution of their larvae described by Ditty *et al*. (2004) for the northern GoM resembles our results, since they also reported a limited presence and low density beyond the continental slope. Likewise, Espinosa-Fuentes and Flores-Coto [1] found lower densities of *C*. *crysos* at stations between the outer shelf and oceanic waters of the sBoC compared to more coastal stations, coinciding with our findings for the south-eastern BoC. On the other hand, *Auxis* spp. is considered mainly neritic [1, 67, 102], but adults and larvae have also been caught in oceanic waters [62, 103]. Espinosa-Fuentes and Flores-Coto [1] found twice the density of *Auxis* spp.’s larvae in the outer shelf and over the slope in comparison with the inner shelf; larvae were absent at coastal stations. These results suggest that *Auxis* spp. spawn over the shelf as well as in oceanic waters in contrast to the neritic *C*. *crysos*, as suggested by Lindo-Atichati *et al*. [33] in the northern GoM and reported by Klawe [103] in the eastern Pacific Ocean, and Matsuura and Sato [104] in southern Brazilian waters.

The presence of larvae of strictly neritic species in the deep-water region is likely explained by their offshore advection by local currents in the BoC, in the north-western gulf and north and east of the Yucatan shelf. Martínez-López and Zavala-Hidalgo [8] describe the seasonal offshore cross-shelf transport and high chl a surface waters off the Tamaulipas-Veracruz (TAVE; western GoM) shelf and in the south-eastern BoC, which is produced by the convergence of seasonal winds along the shelf. This is observed during spring/early summer in TAVE, and in the southern BoC in the fall (see also [45, 105]). Specifically, the higher density of neritic larvae in the deep-water region of the south-eastern BoC during season II, is consistent with the peak river discharges from the Grijalva-Usumacinta riverine system that coincide with offshore advection and high surface chl a concentration in the bay’s central region [8]. The presence of *C*. *crysos* and *Auxis* spp. larvae off the western Yucatan shelf is also consistent with wind-driven westward circulation along the Yucatan shelf and towards the deep waters of the BoC [106] that is intensified during strong wind events that occur in autumn and winter [48]. Once offshore transport occurs, the limited presence and relatively low density of these larvae in the central GoM could also be a result of their quicker development (16 to 40 days; [11–13]), in comparison with mesopelagic larvae, or cumulative mortality if the environmental conditions are unfavourable for their growth and survival [11]. Whether or not these larvae are recruited to favourable nursery habitats is unknown.

Our results clearly show that *C*. *pauciradiatus* is distributed throughout the oceanic waters of the southern gulf. Houde *et al*. [107] and Felder and Camp [64] limited the distribution of *C*. *pauciradiatus* larvae to the north-eastern and north-western GoM, this was likely due to limited surveys in the sGoM. We found significantly greater larval density between April and July. This is consistent with Lamkin’s [74] report of spawning in April in the oceanic waters of the northern GoM. The spawning of *B*. *atlanticus* occurs throughout the year in the Atlantic Ocean and GoM, off the central coast of Brazil [108] and in the Straits of Florida [70]. Zavala-García and Flores-Coto [69] analysed the species’ density based on eight cruises, and observed the highest average density (average of 6 larvae 1000 m^-3^) in August, compared with 2 larvae 1000 m^-3^ in November to December and 1.4 larvae 1000 m^-3^ between March and May. We also found significantly higher densities during season II, which included August (5.8 larvae 1000 m^-3^). Our results and those of Zavala-García and Flores-Coto [69] suggest a spawning peak between August and September. This spawning period overlaps with the transport of high-nutrient river plumes offshore observed in the sGoM from July to September [8], which drive higher productivity and hence food availability for the larvae, likely contributing to their survival.

The lack of significant differences in the seasonal density of the lanternfishes (*B*. *suborbitale* and *N*. *valdiviae*) is consistent with the year-around spawning of the adults [16, 23, 75]. For the northern oceanic GoM, Milligan and Sutton [109] found that larvae of *B*. *suborbitale* and *N*. *valdiviae* had limited variations in density between early summer (April-June) and late summer (July-September). Rodríguez-Varela *et al*. [110] after conducting three cruises between May and July in the Mexican EEZ, also reported that *B*. *suborbitale* was homogeneously distributed in the inner GoM, and *N*. *valdiviae* had higher densities in the BoC, coinciding with the distribution found in our results. Additionally, the broader distribution of larvae from mesopelagic species [111, 112] could be due to the widespread occurrence and high abundance of adults in mesopelagic waters, where environmental conditions are more homogenous [113] compared to surface waters [36, 114]. In addition, adults reproduce and spawn at around 800 m [21, 22] and there is passive horizontal transport during the ascent of eggs and larvae toward the surface and the larval stage is relatively long [23, 24], which might increase the larvae’s distribution.

### Relationship with oceanographic variables

When relating the density of *C*. *crysos* with the oceanographic variables in the GAMs, no explanatory power was obtained. As we hypothesized, neritic larvae that are transported offshore did not show a correlation with environmental variables. The presence of *C*. *crysos* in the deep-water region away from suitable habitat for the larvae will likely lead to the loss of those individuals. However, further research is needed to determine their fate.

The relationship between the oceanographic variables and larval density observed in the response plots from the GAMs for *Auxis* spp., *B*. *atlanticus*, *C*. *pauciradiatus*, *B*. *suborbitale* and *N*. *valdiviae* were very similar (e.g., higher density at high SSTs or high surface chl a concentrations). Surface chl a concentration, WS and SST (in order of their explanatory power in most models), showed to have an impact on habitat suitability, which is often interlinked [115, 116] with the survival of the early life stages of fish (see [31, 117]).

Surface chl a concentration is usually used as a proxy for phytoplankton biomass and indicator of food availability [82, 86], and higher chl a concentrations are commonly found at higher WS [118], lower stratification [119] and lower SSTs [120]. Despite the significantly positive correlation between chl a and the density observed for *Auxis* spp. (season I and II), *B*. *atlanticus* and *C*. *pauciradiatus* (season I) and *B*. *suborbitale* (season II), most of the observations of larval presence were found at low concentrations (0.05 and 0.12 mg m^-3^), typical of the deep-water region. Nevertheless, the positive relationship may indicate a higher prey availability for the larvae, which could lead to faster growth and survival and greater density [117]. In the GoM, higher chl a concentrations may be associated with fronts generated by the interaction between cyclonic and LCEs and AC eddies in the central GoM [8, 41, 105], as well the semi-permanent CE that is characteristic of the BoC [36], or due to wind-driven seasonal upwelling in the western and southern shelves of the GoM [105] and in the western Yucatan shelf [106].

The overall shape of the additive effect response plots of WS and density exhibited greater variation among models compared with other variables. In season I, the relationship between density and WS varied between species, and greater variability in density was observed at higher WS values (> 7 m s^-1^), while in season II, WS did not exceed 6 m s^-1^ and the relationship with larval density was generally positive. According to Mackenzie and Kiørboe [89] and Kloppmann *et al*. [90], high WS drives mixing in the surface layer and increases turbulence [115, 116], favouring primary production, supporting an increase in zooplankton biomass and increasing the encounter rate between the larvae and their prey. However, WS that are too high will decrease the feeding success of larvae, as the prey capture success decreases [121, 122]. Our results showed that most of the larval observations during both seasons were found at intermediate WS (Season I: 4.62 ± 1.64 m s^-1^; Season II: 4.01 ± 1.45 m s^-1^). These results are consistent with Lasker’s [123] “stable ocean” hypothesis, in which he proposed that strong winds can negatively influence larval feeding success.

The spatial variation in SSTs in the GoM’s deep-water region is small [50] compared to what is observed over the continental shelf [45, 106]. In this study, a positive relationship between SST and larval density was found in every species. Most of the observations and higher densities in season I were between 28 and 30°C (63%), and 29.5 to 31°C for season II (90%), with few observations in either colder or warmer waters. According to Fuiman and Werner [31], fish growth rates are typically highest at intermediate temperatures within their environmental tolerance scope, which may be reflected in higher densities due to lower cumulative mortality. However, temperature-dependent growth rates of our target species are lacking, and SSTs do not reflect the vertical thermal structure of the water column, therefore future studies should examine the relationship between larval growth rates and in situ temperature.

Our results indicated that the distribution of larvae and higher densities were largely restricted to stations with low stratification values; 63% of the observations were between 1100 and 1700 J. Studies such as Franco-Gordo *et al*. [124] for the central Pacific coast of Mexico or Moyano and Hernández-León [119] along the Gran Canaria Island shelf have reported that stratification, which is driven primarily by SST and WS (see [125–127]), can define the distribution of larvae close to the continental slope. Lower stratification is usually related to greater WS and mixing in the surface layer, which can provide nutrients to the euphotic zone [84, 85] yielding greater food availability, hence higher larval survival and recruitment [31].

The remaining variables considered in this study (depth, SSH and salinity) were retained in only one model and had a low influence in the final explained deviance. For example, the limited relationship between bottom depth and *C*. *pauciradiatus’* density in season I is reasonable for a mesopelagic species found basin-wide, which coincides with the wide adult distribution [64, 128]. Likewise, the small correlation between SSH and *C*. *pauciradiatus*’ density in season I and *N*. *valdiviae*’s in season II, reflect the oceanic habitat that adults of these species occupy and in which they spawn [16, 64, 74]. Additionally, the highest abundances were found at intermediate SSH values for mesopelagic species, such as *C*. *pauciradiatus* suggesting that adults can be found and spawn at frontal environments (e.g., in regions of interaction between AC and CE), where an increased concentration of prey can be found [128]. Mean salinity was only significantly related with *B*. *suborbitale* in season I, with a steep decrease in density at salinities higher than 36.55 psu. These salinities are toward the highest values reported for surface waters of the GoM [129, 130], and according to Fuiman and Werner [31] salinity influences the development of fish larvae, although to a lesser extent than temperature.

## Conclusions

In this study, we highlight the importance of the contrasting early life stages of fish and their relationship with oceanographic variables for a better understanding of the distribution and density patterns of larvae in the southern GoM’s deep-water region. The GAMs allowed us to examine how the same oceanographic conditions related differently with the density of larvae. In addition, analysing each species model provided further insight in the suitable habitat conditions of larvae in species with contrasting life histories.

Pooling several cruises into a pre-defined season might conceal the relationship between specific oceanographic phenomena and species-specific larval fish density. However, it provides (1) a more integrative description of larval distribution and variations in density between seasons, and (2) a more robust examination of how the densities of different species are related to a uniform set of oceanographic variables. Further studies might consider encompassing both the continental shelf and the deep-water region to describe the distribution gradients in species that occupy the whole basin, and exploring the consequences of finding the larvae of neritic species in the deep-water region. Our results will provide a useful baseline for future studies that delimit the potential habitat distribution under the same oceanographic conditions and for evaluating the impact of climate change on pelagic ecosystems.

## Supporting information

S1 Table GAMs using season as a categorical variable and as a categorical smoothing term. Season I (April-July) in red and season II (August-October) in blue.
(DOCX)

S1 Fig Response plots of the oceanographic variables’ additive effect on the density of target species. Season I (April-July) in red and season II (August-October) in blue. Smoothed values represented by a continuous line, and shaded color indicates 95% confidence intervals. Continuation.
(DOCX)

S2 Fig Scatterplots of species density and oceanographic variables. Values are represented without transformation. Season I (April-July) in red and season II (August-October) in blue.
(DOCX)
