# Supplementary material for: Distribution and densities of fish larvae species with contrasting life histories as a function of oceanographic variables in the deep-water region of the southern Gulf of Mexico
Source: PLoS One. 2023 Feb 13;18(2):e0280422. doi: 10.1371/journal.pone.0280422 (PMC9925083; doi:10.1371/journal.pone.0280422)
Supplement: S1 Table — Season I (April-July) in red and season II (August-October) in blue. (DOCX) [file pone.0280422.s001.docx]

# **SUPPORTING INFORMATION**

**S1 table: GAMs using season as a categorical variable and as a categorical smoothing term**. Season I (April-July) in red and season II (August-October) in blue.

| ***Auxis* spp.** | | | | |
| --- | --- | --- | --- | --- |
| Family: Tweedie(p=1.368) | | Link function: log | | |
|  | edf | Ref.df | F | p-value |
| s(Stratification):seasonT1 | 1.73 | 1.92 | 1.14 | 0.31 |
| s(Stratification):seasonT2 | 1.00 | 1.00 | 10.73 | 0.001173 ** |
| s(SST):seasonT1 | 2.22 | 2.61 | 1.87 | 0.079772 . |
| s(SST):seasonT2 | 1.00 | 1.00 | 8.02 | 0.004867 ** |
| s(SSH):seasonT1 | 1.00 | 1.00 | 4.08 | 0.044395 * |
| s(SSH):seasonT2 | 1.00 | 1.00 | 0.47 | 0.49 |
| s(Chl):seasonT1 | 1.55 | 1.80 | 4.37 | 0.019704 * |
| s(Chl):seasonT2 | 1.91 | 1.99 | 7.72 | 0.000885 *** |
| s(wind_speed):seasonT1 | 1.50 | 1.74 | 8.43 | 0.000450 *** |
| s(wind_speed):seasonT2 | 1.91 | 1.99 | 13.00 | 3.15e-06 *** |
| Signif. codes: 0 ‘***’ 0.001 ‘**’ 0.01 ‘*’ 0.05 ‘.’ 0.1 ‘ ’ 1 | | | | |
| R-sq.(adj) = 0.282 Deviance explained = 39.9% | | | | |
| -REML = 462.25 Scale est. = 9.5799 n = 326 | | | | |
| ***Benthosema suborbitale*** | | | | |
| Family: Tweedie(p=1.331) | | Link function: log | | |
|  | edf | Ref.df | F | p-value |
| s(Stratification):seasonT1 | 1.31 | 1.53 | 6.33 | 0.020863 * |
| s(Stratification):seasonT2 | 1.85 | 1.97 | 4.03 | 0.030785 * |
| s(S_mean_0_200):seasonT1 | 1.84 | 1.97 | 5.86 | 0.002496 ** |
| s(S_mean_0_200):seasonT2 | 1.67 | 1.88 | 0.83 | 0.37 |
| s(SST):seasonT1 | 1.87 | 1.98 | 8.54 | 0.000478 *** |
| s(SST):seasonT2 | 1.84 | 1.97 | 3.47 | 0.026782 * |
| s(Chl):seasonT1 | 1.00 | 1.00 | 0.35 | 0.56 |
| s(Chl):seasonT2 | 2.55 | 2.85 | 9.13 | 2.66e-05 *** |
| s(wind_speed):seasonT1 | 1.51 | 1.75 | 4.70 | 0.008301 ** |
| s(wind_speed):seasonT2 | 1.52 | 1.76 | 5.37 | 0.004629 ** |
| Signif. codes: 0 ‘***’ 0.001 ‘**’ 0.01 ‘*’ 0.05 ‘.’ 0.1 ‘ ’ 1 | | | | |
| R-sq.(adj) = 0.202 Deviance explained = 27.1% | | | | |
| -REML = 942.19 Scale est. = 6.7079 n = 326 | | | | |
| ***Bregmaceros atlanticus*** | | | | |
| Family: Tweedie(p=1.338) | | Link function: log | | |
|  | edf | Ref.df | F | p-value |
| s(Stratification):seasonT1 | 1.00 | 1.00 | 13.11 | 0.000341 *** |
| s(Stratification):seasonT2 | 1.00 | 1.00 | 0.00 | 0.97 |
| s(SST):seasonT1 | 1.00 | 1.00 | 16.06 | 7.66e-05 *** |
| s(SST):seasonT2 | 1.00 | 1.00 | 1.57 | 0.21 |
| s(Chl):seasonT1 | 1.00 | 1.00 | 10.31 | 0.001462 ** |
| s(Chl):seasonT2 | 1.86 | 1.98 | 4.92 | 0.013589 * |
| s(wind_speed):seasonT1 | 1.00 | 1.00 | 1.90 | 0.17 |
| s(wind_speed):seasonT2 | 2.58 | 2.85 | 4.26 | 0.005362 ** |
| Signif. codes: 0 ‘***’ 0.001 ‘**’ 0.01 ‘*’ 0.05 ‘.’ 0.1 ‘ ’ 1 | | | | |
| R-sq.(adj) = 0.104 Deviance explained = 17.3% | | | | |
| -REML = 724.99 Scale est. = 6.4673 n = 326 | | | | |
| ***Cubiceps pauciradiatus*** | | | | |
| Family: Tweedie(p=1.335) | | Link function: log | | |
|  | edf | Ref.df | F | p-value |
| s(Stratification):seasonT1 | 2.35 | 2.67 | 4.40 | 0.00436 ** |
| s(Stratification):seasonT2 | 2.40 | 2.75 | 3.10 | 0.07599 . |
| s(S_mean_0_200):seasonT1 | 1.92 | 1.99 | 5.69 | 0.00289 ** |
| s(S_mean_0_200):seasonT2 | 1.66 | 1.87 | 2.94 | 0.03616 * |
| s(SST):seasonT1 | 1.00 | 1.00 | 10.76 | 0.00116 ** |
| s(SST):seasonT2 | 1.80 | 1.97 | 6.88 | 0.00082 *** |
| s(SSH):seasonT1 | 1.22 | 1.39 | 8.02 | 0.00238 ** |
| s(SSH):seasonT2 | 1.00 | 1.00 | 3.76 | 0.05335 . |
| s(Chl):seasonT1 | 1.65 | 1.88 | 4.73 | 0.02040 * |
| s(Chl):seasonT2 | 1.64 | 1.86 | 0.81 | 0.47094 |
| s(wind_speed):seasonT1 | 2.08 | 2.46 | 2.80 | 0.07786 . |
| s(wind_speed):seasonT2 | 2.10 | 2.46 | 18.09 | < 2e-16 *** |
| Signif. codes: 0 ‘***’ 0.001 ‘**’ 0.01 ‘*’ 0.05 ‘.’ 0.1 ‘ ’ 1 | | | | |
| R-sq.(adj) = 0.217 Deviance explained = 49.6% | | | | |
| -REML = 635.89 Scale est. = 9.6242 n = 326 | | | | |
| ***Notolychnus valdiviae*** | | | | |
| Family: Tweedie(p=1.375) | | Link function: log | | |
|  | edf | Ref.df | F | p-value |
| s(Stratification):seasonT1 | 1.00 | 1.00 | 7.99 | 0.00498 ** |
| s(Stratification):seasonT2 | 1.00 | 1.00 | 0.28 | 0.5961 |
| s(SST):seasonT1 | 2.79 | 2.96 | 3.93 | 0.01023 * |
| s(SST):seasonT2 | 1.00 | 1.00 | 0.23 | 0.63153 |
| s(wind_speed):seasonT1 | 2.57 | 2.86 | 7.84 | 5.98e-05 *** |
| s(wind_speed):seasonT2 | 1.17 | 1.31 | 3.39 | 0.07211 . |
| s(Lon, Lat) | 12.75 | 17.16 | 4.27 | < 2e-16 *** |
| Signif. codes: 0 ‘***’ 0.001 ‘**’ 0.01 ‘*’ 0.05 ‘.’ 0.1 ‘ ’ 1 | | | | |
| R-sq.(adj) = 0.23 Deviance explained = 32.8% | | | | |
| -REML = 1186.6 Scale est. = 5.3505 n = 326 | | | | |
