# Supplementary material for: Distribution and densities of fish larvae species with contrasting life histories as a function of oceanographic variables in the deep-water region of the southern Gulf of Mexico
Source: PLoS One. 2023 Feb 13;18(2):e0280422. doi: 10.1371/journal.pone.0280422 (PMC9925083; doi:10.1371/journal.pone.0280422)

**S1 Fig. Response plots of the oceanographic variables’ additive effect on the density of target species.** Season I (April-July) in red and season II (August-October) in blue. Smoothed values represented by a continuous line, and shaded color indicates 95% confidence intervals.


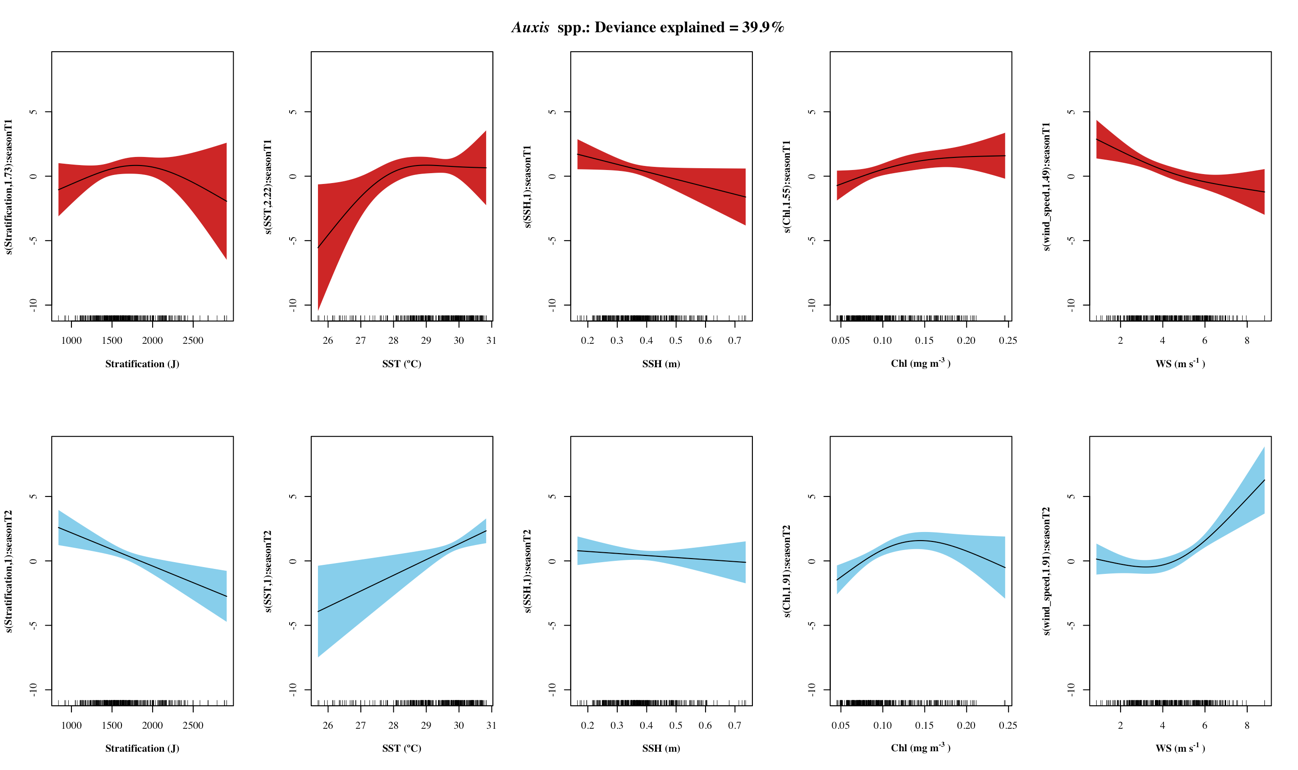


**S1 Fig. Response plots of the oceanographic variables’ additive effect on the density of target species.** Season I (April-July) in red and season II (August-October) in blue. Smoothed values represented by a continuous line, and shaded color indicates 95% confidence intervals. Continuation.


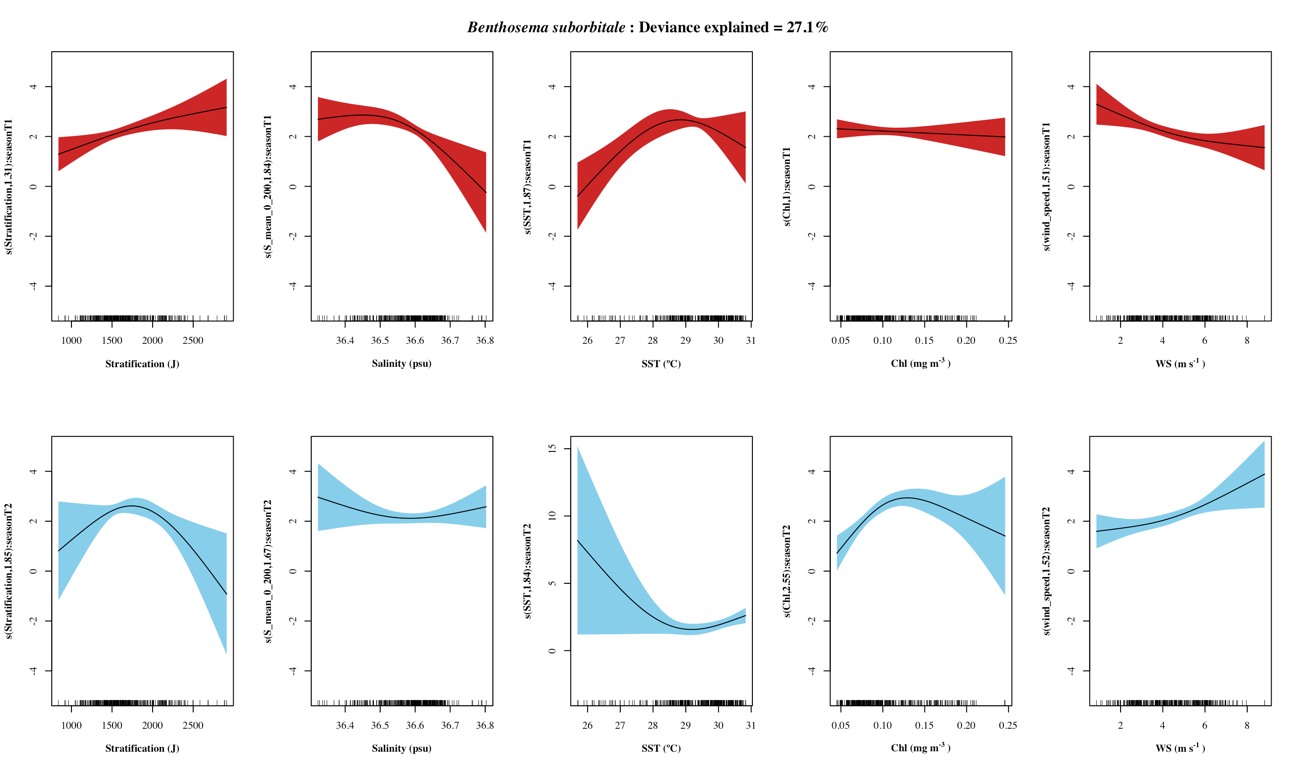


**S1 Fig. Response plots of the oceanographic variables’ additive effect on the density of target species.** Season I (April-July) in red and season II (August-October) in blue. Smoothed values represented by a continuous line, and shaded color indicates 95% confidence intervals. Continuation.


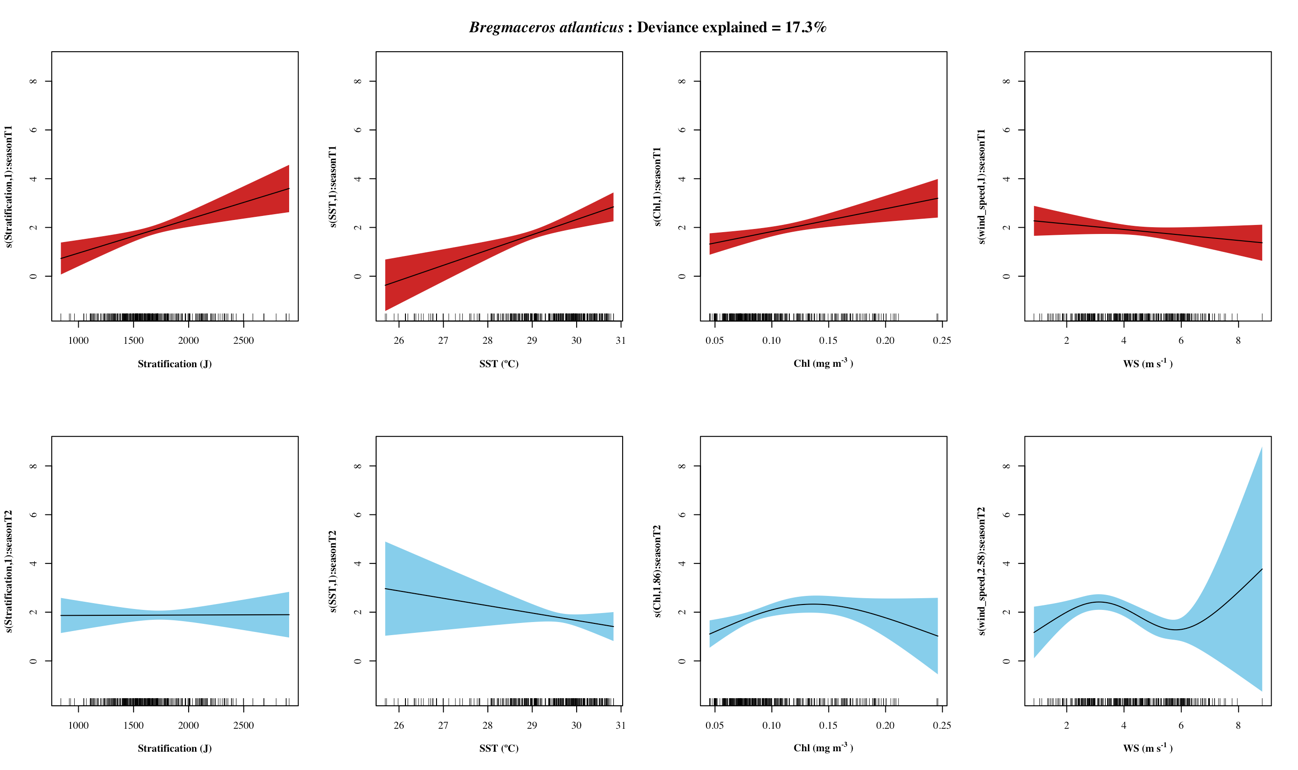


**S1 Fig. Response plots of the oceanographic variables’ additive effect on the density of target species.** Season I (April-July) in red and season II (August-October) in blue. Smoothed values represented by a continuous line, and shaded color indicates 95% confidence intervals. Continuation.


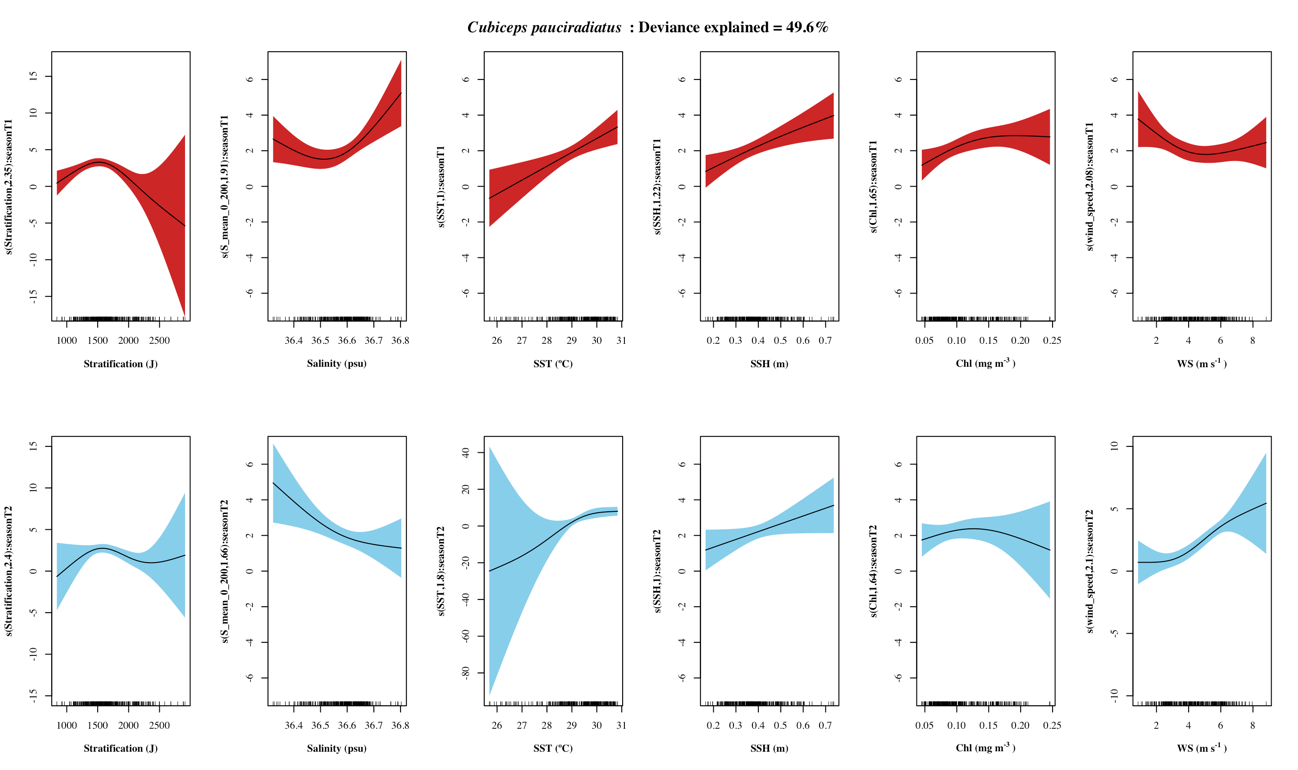


**S1 Fig. Response plots of the oceanographic variables’ additive effect on the density of target species.** Season I (April-July) in red and season II (August-October) in blue. Smoothed values represented by a continuous line, and shaded color indicates 95% confidence intervals. Continuation.


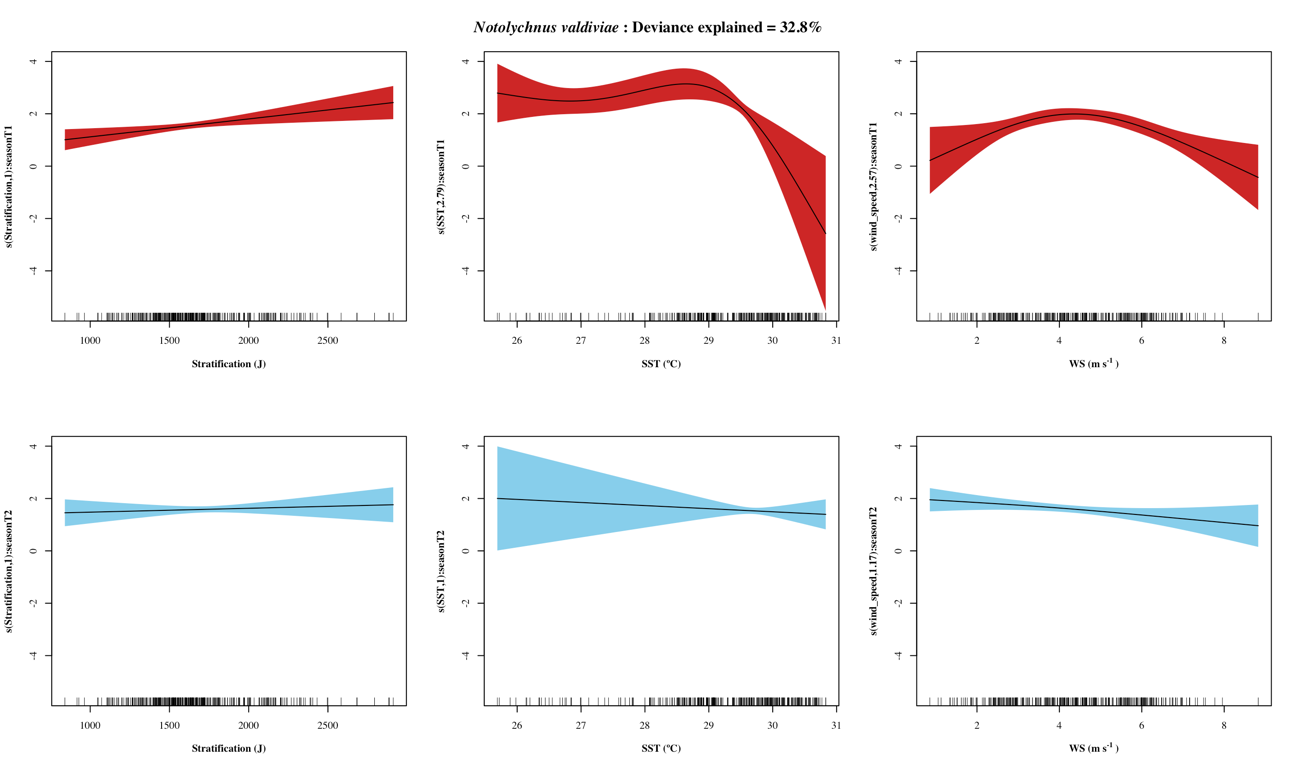

Supplement: S1 Fig — Season I (April-July) in red and season II (August-October) in blue. Smoothed values represented by a continuous line, and shaded color indicates 95% confidence intervals. Continuation. (DOCX) [file pone.0280422.s002.docx]
