# Supplementary material for: Distribution and densities of fish larvae species with contrasting life histories as a function of oceanographic variables in the deep-water region of the southern Gulf of Mexico
Source: PLoS One. 2023 Feb 13;18(2):e0280422. doi: 10.1371/journal.pone.0280422 (PMC9925083; doi:10.1371/journal.pone.0280422)
Supplement: S2 Fig — Values are represented without transformation. Season I (April-July) in red and season II (August-October) in blue. (DOCX) [file pone.0280422.s003.docx]

**S2 Fig: Scatterplots of species standardized abundance and oceanographic variables.** Red (blue) colors represent season I: April-July (season II: August-October).

**
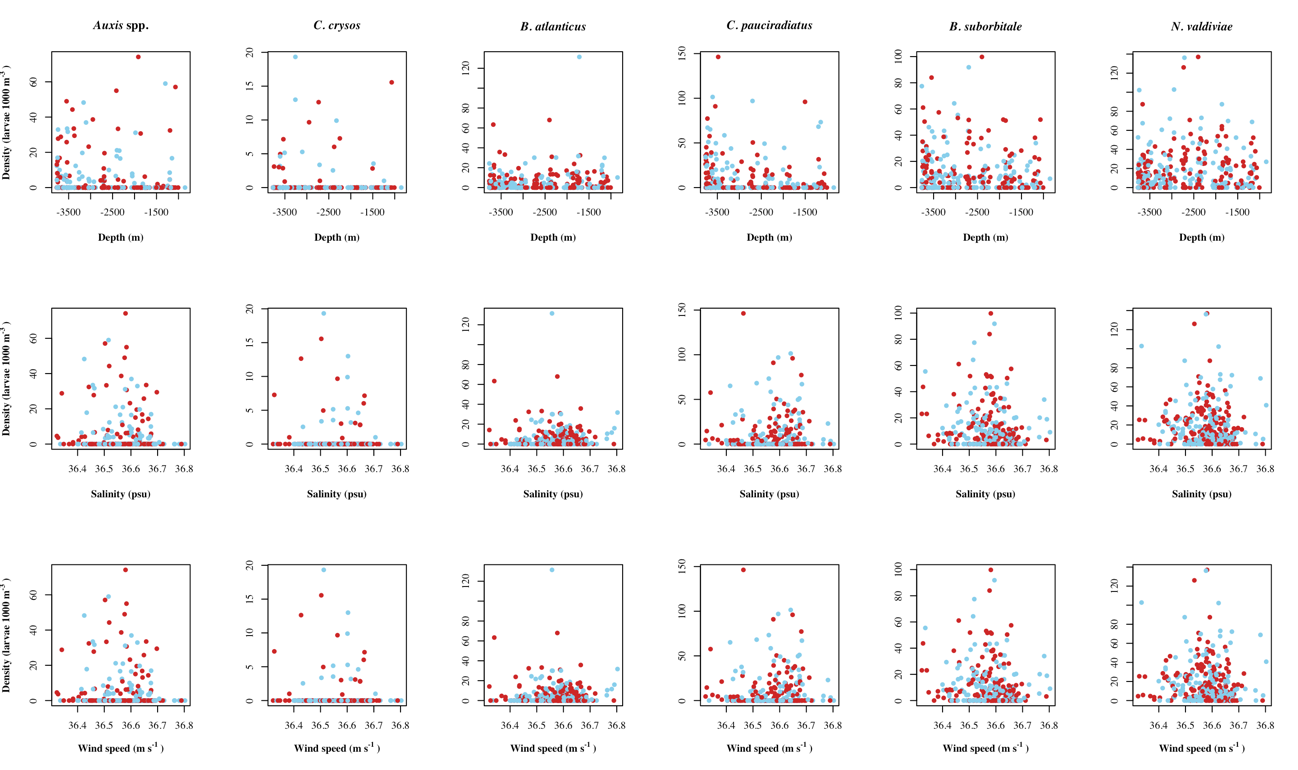
**

**S2 Fig: Scatterplots of species standardized abundance and oceanographic variables.** Red (blue) colors represent season I: April-July (season II: August-October). Continuation.

**
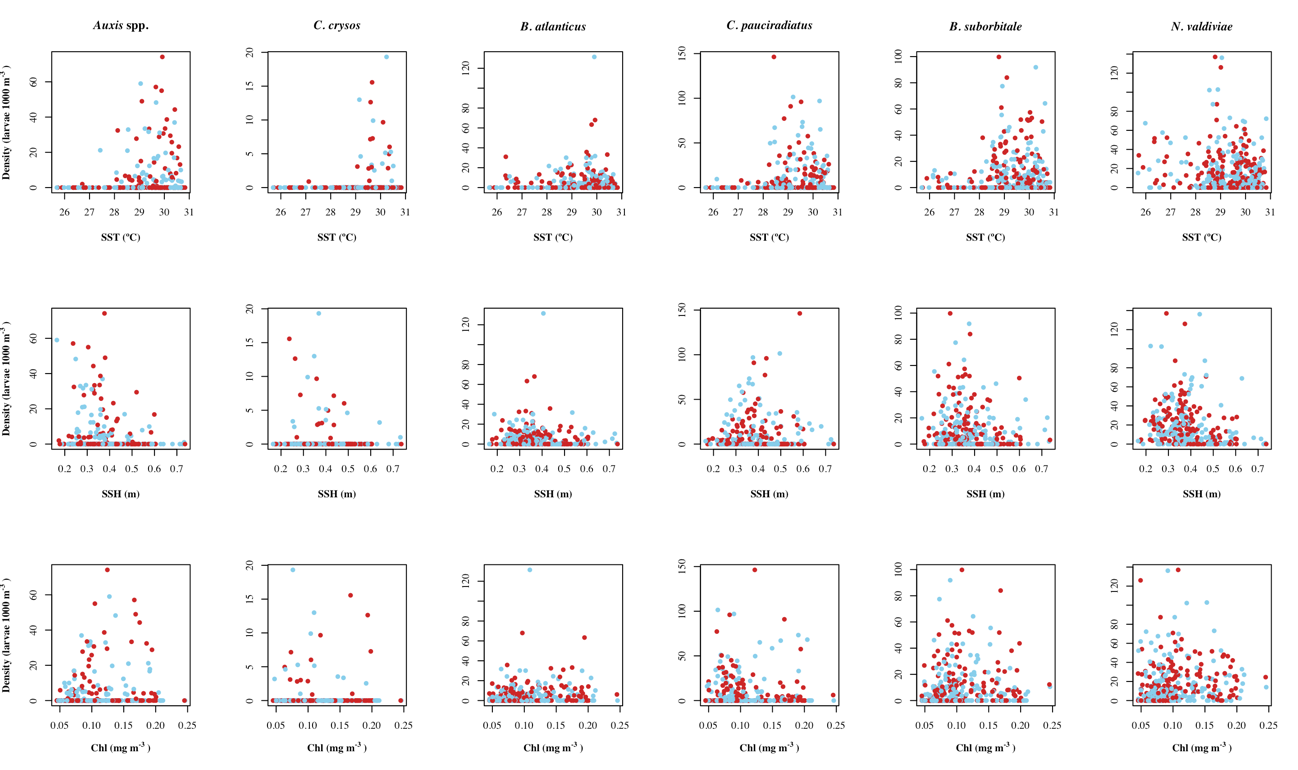
**

**S2 Fig: Scatterplots of species standardized abundance and oceanographic variables.** Red (blue) colors represent season I: April-July (season II: August-October). Continuation.

**
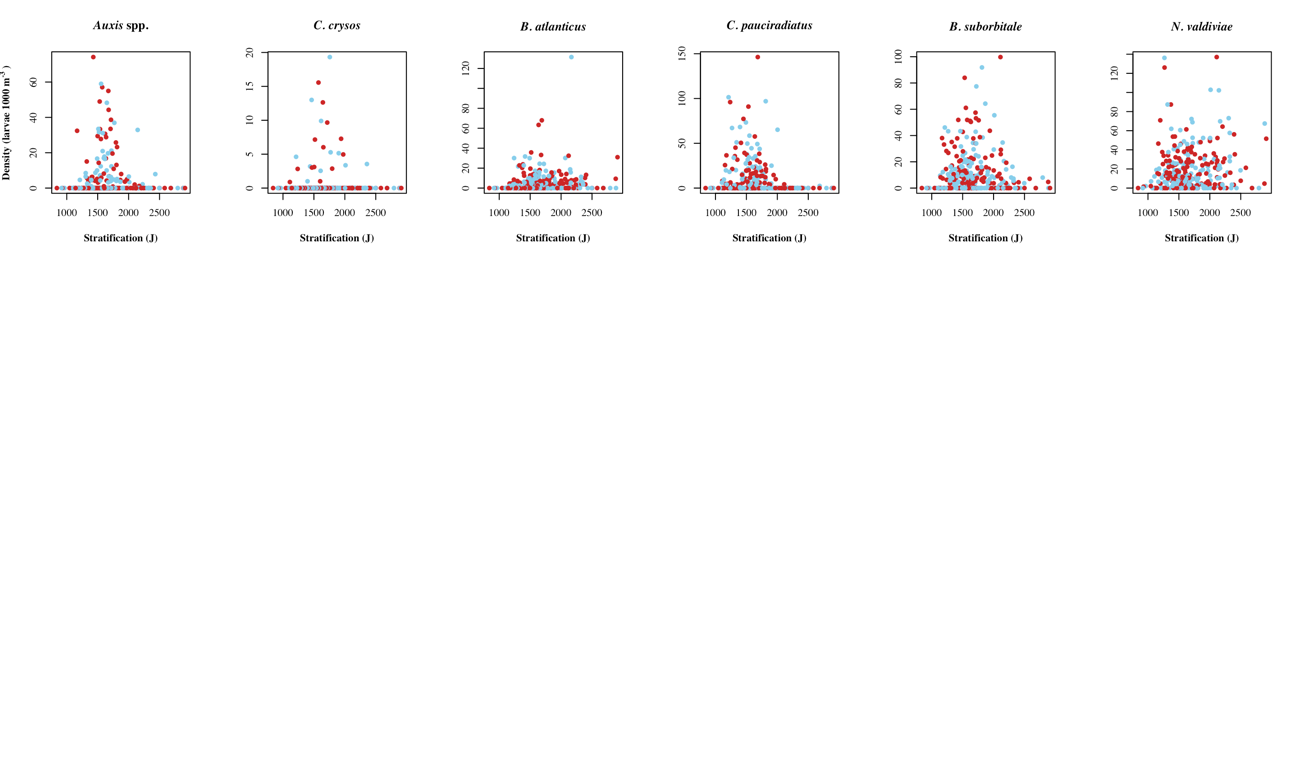
**
